# Delineation of hypoxia-induced proteome shifts in osteosarcoma cells with different metastatic propensities

**DOI:** 10.1038/s41598-019-56878-x

**Published:** 2020-01-20

**Authors:** Zifeng Song, Martin C. Pearce, Yuan Jiang, Liping Yang, Cheri Goodall, Cristobal L. Miranda, Milan Milovancev, Shay Bracha, Siva K. Kolluri, Claudia S. Maier

**Affiliations:** 1 Department of Chemistry, Oregon State University, Oregon, USA; 2 Department of Environmental & Molecular Toxicology, Oregon State University, Oregon, USA; 3 Department of Statistics, Oregon State University, Oregon, USA; 4 College of Veterinary Medicine, Oregon State University, Oregon, USA; 5 Linus Pauling Institute, Oregon State University, Oregon, USA

**Keywords:** Proteomics, Bone cancer

## Abstract

Osteosarcoma (OS) is the most common bone cancer in children and young adults. Solid tumors are characterized by intratumoral hypoxia, and hypoxic cells are associated with the transformation to aggressive phenotype and metastasis. The proteome needed to support an aggressive osteosarcoma cell phenotype remains largely undefined. To link metastatic propensity to a hypoxia-induced proteotype, we compared the protein profiles of two isogenic canine OS cell lines, POS (low metastatic) and HMPOS (highly metastatic), under normoxia and hypoxia. Label-free shotgun proteomics was applied to comprehensively characterize the hypoxia-responsive proteome profiles in the OS cell phenotypes. Hypothesis-driven parallel reaction monitoring was used to validate the differential proteins observed in the shotgun data and to monitor proteins of which we expected to exhibit hypoxia responsiveness, but which were absent in the label-free shotgun data. We established a “distance” score (|*z*_*HMPOS*_ − *z*_*POS*_|), and “sensitivity” score (|*z*_*Hypoxia*_ − *z*_*Normoxia*_) to quantitatively evaluate the proteome shifts exhibited by OS cells in response to hypoxia. Evaluation of the sensitivity scores for the proteome shifts observed and principal component analysis of the hypoxia-responsive proteins indicated that both cell types acquire a proteome that supports a Warburg phenotype with enhanced cell migration and proliferation characteristics. Cell migration and glucose uptake assays combined with protein function inhibitor studies provided further support that hypoxia-driven adaption of pathways associated with glycolytic metabolism, collagen biosynthesis and remodeling, redox regulation and immunomodulatory proteins typify a proteotype associated with an aggressive cancer cell phenotype. Our findings further suggest that proteins involved in collagen remodeling and immune editing may warrant further evaluation as potential targets for anti-metastatic treatment strategies in osteosarcoma.

## Introduction

Osteosarcoma (OS) is the most common bone cancer in children and young adults, affecting around 400 children in the US every year^1,2^. OS is a malignant neoplasm of the bone having a high propensity for pulmonary metastasis with approximately 20% of patients diagnosed with metastatic tumors and 80% developing metastasis despite standard of care treatment including surgery and chemotherapy^3,4^. Less than 15% of patients with metastatic OS experience 5-year survival rates with current non-targeted treatments, and, over the last three decade there was little improvement in survival rates^5–8^. Thus, there is an urgent need for new therapeutic strategies aiming to control metastasis in OS. We aimed to address this urgency by applying a proteome-centered discovery approach to identify protein targets that can be used as markers in tumor phenotyping or as leads for treatment strategies.

The hypoxic tumor microenvironment functions as the dominant driving force for cancer progression, drug resistance, development of metastatic potential, and overall poor clinical outcomes^9–12^. Therefore, the identification of hypoxia-inducible protein targets responsible for cancer progression, malignant transformation and metastasis is needed for deepening our understanding of OS biology. Although proteomic studies have been carried out to identify hypoxia-responsive proteins and associated molecular pathways in diverse cancer cell types^13–15^, similar studies for osteosarcoma cells are sparse^6,16^. Because mechanistic insights found in one type of cancer are not transferable to other cancer types, we set out to identify hypoxia–responsive proteins in OS phenotypes and their roles in contributing to aggressive OS phenotypes^17^.

To explore the impact of the cell phenotype on the hypoxia-responsive proteome we chose two isogenic canine OS cell lines with different phenotypes: POS (primary origin) displaying a low metastatic phenotype and HMPOS (metastatic origin) exemplifying a highly metastatic phenotype^18^. We used a combination of label-free quantitative proteomics and parallel reaction monitoring (PRM) mass spectrometry for assessing protein expression changes induced by hypoxia in both OS cell phenotypes. Biochemical assays confirmed the functional significance of the identified hypoxia-responsive protein targets and pathways associated with cell migration and aggressiveness. We believe that the findings of this protein-centric study hints to potential strategies to block cell migration and metastatic spread of osteosarcoma.

## Results

### Shotgun proteomics for comparative evaluation of hypoxia-induced proteome shifts in osteosarcoma cells with different metastatic propensities

**Figure 1:**
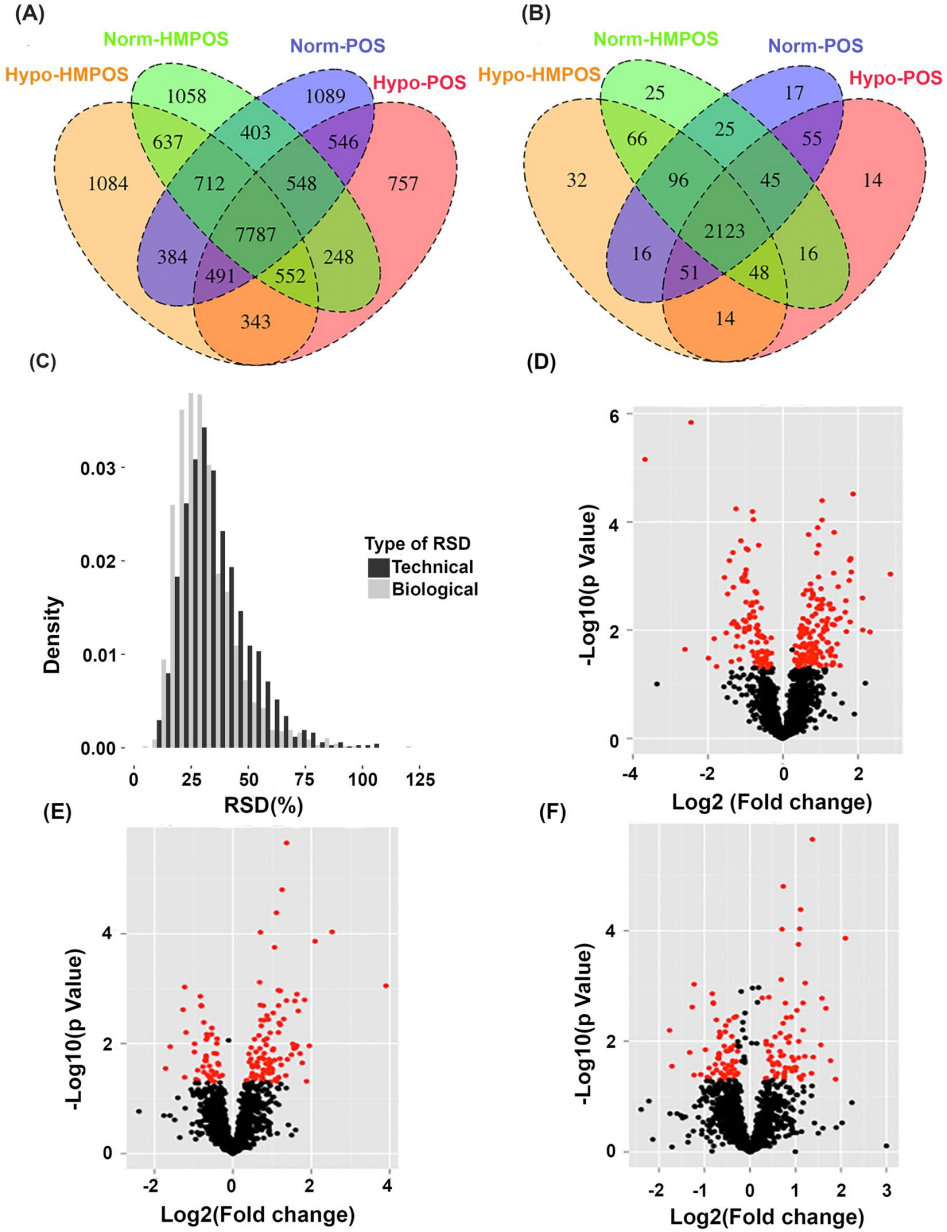
Hypoxia remodels the osteosarcoma proteome in a cell-type specific manner. Venn diagrams for the distribution of identified peptides (**A**) and proteins (**B**) for all four groups; (**C**) Histograms for technical and biological RSD distributions of identified proteins; (**D**–**F**) Volcano plots illustrating the distributions of *p*-values and fold changes of all 1704 proteins that were consistently found among the 4 groups. (**D**) Norm-HMPOS versus Norm-POS; (**E**) Hypo-POS versus Norm-POS; (**F**) Hypo-HMPOS versus Norm-HMPOS. The −log_10_ (*p*-Value) is plotted against log_2_ (Fold Change). Pair-wise comparison data in Supplemental Table 3(a–c) were used for generating the volcano plots. The red dots represent proteins that pass statistical threshold set at *p*-value < 0.05 and fold-change > ±1.2.

The findings of our comparative shotgun proteomic studies are summarized in Fig. 1. A total of 17186 peptides associated with 2653 proteins (Supplemental Table S1) were identified with 1% false discovery rate (FDR) at both peptide and protein levels across all four groups (Fig. 1A,B). More than 1700 proteins (Supplemental Table S2) that were consistently found across all four groups, were selected for further statistical analysis. The analysis of the relative standard deviation (RSD) distributions of these proteins (Fig. 1C) reveals that more than 82% of the consistently found proteins were determined with technical RSDs < 50%. The filtered label-free data sets were fitted by a linear regression (LR) model described in the method section. Fold changes ≥ ±1.2 and *p*-values < 0.05 were set as the threshold for significant differentially expressed proteins^19,20^. It is noteworthy that we chose the *p*-value threshold at 0.05 without adjusting for multiple comparisons initially to allow more findings due to the discovery nature of the label-free proteomic study. Figure 1 shows volcano plots of the following pair-wise comparisons: (D) Norm-HMPOS versus Norm-POS to define the cell-type specific proteins; (E) Hypo-POS versus Norm-POS to determine the impact of hypoxia on the POS-specific proteome and (F) Hypo-HMPOS versus Norm-HMPOS to identify the hypoxia–responsive proteins in the HMPOS cells.

### OS cell-type specific proteomes under normoxia

We next set out to identify proteins associated with metastatic potential by comparing the proteomic profiles of the two isogenic cell lines, POS and HMPOS^21^, cultured under normoxia. We found 248 proteins that were differentially expressed**;** 155 proteins were up-regulated and 93 down-regulated in HMPOS cells compared to POS cells (Fig. 1D and Supplementary Table S3a). The up-regulated and down-regulated proteins were mapped separately to canonical pathways (Supplementary Table S3a). Pathways enriched in the up-regulated protein group were cell cycle control of chromosomal replication, phagosome maturation, glycolysis and lipid metabolisms. The down-regulated proteins were associated with proteins kinase A signaling, TCA cycle I and actin cytoskeleton signaling. The top molecular and cellular functions were associated with cellular movement, cell death, and survival (Supplementary Summaries S1a,b). Overall, HMPOS cells displayed a proteome consistent with the Warburg effect indicative of a more aggressive cancerous phenotype compared to the low metastatic POS cells under normoxia^22,23^.

### Hypoxia induced proteome changes in the parental and highly metastatic OS cells

Next, we were interested in determining if hypoxia differently impacts the proteome of the low metastatic parental cells, POS, compared to the highly metastatic cells, HMPOS. We extracted proteins with *p*-values < 0.05 for the hypoxia coefficient in the LR model and fold changes ≥ 1.2, which resulted in 145 hypoxia-responsive proteins in POS and 131 hypoxia-responsive proteins in HMPOS cells. Ingenuity Pathway Analysis results have been compiled in Supplementary Table S3b,c. Function annotations were performed for hypoxia-responsive proteins. Hypoxia stimulated the expression of proteins associated with carbohydrate metabolism, synthesis of reactive oxygen species, cellular growth and proliferation, and cellular movement in both cell types (Fig. S1A,B). In contrast, apoptosis was inhibited in both POS and HMPOS cells when exposed to hypoxia.

**Figure 2:**
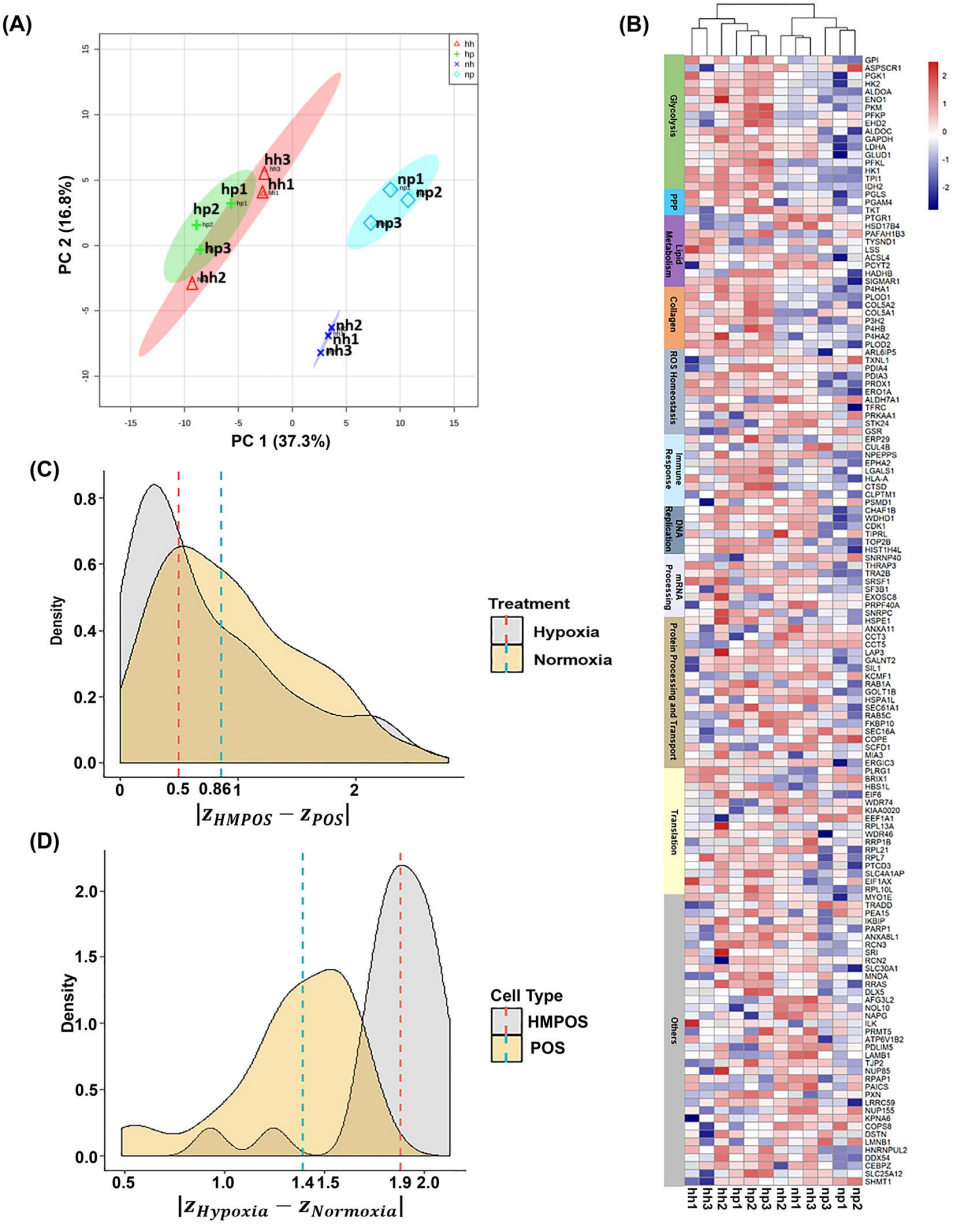
Proteome shifts and sensitivities to hypoxia dependent on the metastatic phenotype of the OS cells. (**A**) Score plot from the principal component analysis (PCA) of the hypoxia responsive proteins (p-value < 0.05 for Treatment term in the LR model) plots; (**B**) Heat map shows hypoxia-responsive proteins with statistically significant changes in expression levels after LR test. Columns indicate the biological samples, and rows indicate proteins. Protein intensities were normalized by *z*-scores, which were color-coded as indicated. Hierarchical clustering was performed using Euclidean distances between biological samples. Data used for the generation of the heat map and cluster analysis are compiled in the Supplemental Table S5. Abbreviations used: hh, Hypo-HMPOS; hp, Hypo-POS; nh, Norm-HMPOS; np, Norm-POS. (**C**) Density plot shows the distributions of z-score subtraction (|${z}_{HMPOS}-{z}_{POS}$|), which indicates the degree of differences in the expression pattern of hypoxia-responsive proteins between POS and HMPOS cells under hypoxia and normoxia; (**D**) Density plots show the distributions of the sensitivity scores ($|{z}_{Hypoxia}-{z}_{Normoxia}|$) for proteins of POS and HMPOS cells, suggesting differential sensitivities of POS and HMPOS cells in response to hypoxic tensions; normalized z-scores of hypoxia-responsive protein targets were calculated based on PRM assay data using extracted ion chromatograms for the proteins and considering all four groups.

To evaluate similarities and differences in the proteomes in both cell types we used principal component analysis for multivariate pattern recognition analysis on our quantitative proteome datasets. A clear separation between the hypoxia datasets (Hypo-POS and Hypo-HMPOS) and normoxia datasets (Norm-POS and Norm-HMPOS) was observed, with PC1 37.3% and PC2 16.8% (Fig. 2A). However, interestingly, the proteomic datasets obtained for the POS and HMPOS samples partially merged under hypoxia, suggesting proteome adaption and remodeling under hypoxia. These adaptive changes in the proteomes were also apparent in the heat map (Fig. 2B) which visualizes the relative expression levels of hypoxia-responsive proteins in biological replicates of POS and HMPOS cells. For instance, the hypoxia-induced upregulation of glycolytic proteins, collagen biosynthesis and redox associated and immune function proteins is observable for both cell lines. To further quantitatively assess the hypoxia-induced proteome shift, protein abundances were normalized as z-scores, and the absolute subtraction of z-scores between HMPOS proteins and POS proteins (|${z}_{HMPOS}-{z}_{POS}$|) was defined as “distance”, which can be used to measure the similarities/differences in the protein expression. The distribution of the distance (z-score subtractions) between HMPOS and POS proteins under hypoxia and normoxia was visualized as separate density plots. As shown in Fig. 2C, a left shift in the median of distances can be observed from 0.86 under normoxia toward 0.50 under hypoxia. This suggests that hypoxia drives POS and HMPOS cells to remodel their proteomes resulting in similar proteotypes.

**Figure 3:**
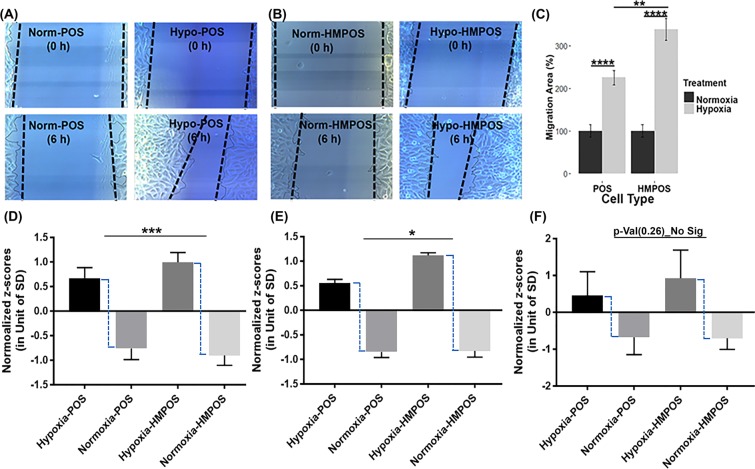
Hypoxia promotes migration of POS and HMPOS cells (**A**,**B**) Images of wound-healing assays of POS and HMPOS cells treated with hypoxia for 6 hours; (**C**) Stimulatory effect of hypoxia on the migration abilities of POS cells and HMPOS cells; The z-scores of hypoxia-responsive protein targets were clustered and visualized separately based on their involvements in cellular pathways, such as (**D**) glycolysis/PPP, (**E**) collagen biosynthesis, and (**F**) ROS homeostasis Error bars, ±S.E.M. *p < 0.05, ***p < 0.001, **p < 0.01, ****p < 0.0001.

This hypoxia-induced shift in proteome biology suggests OS cell phenotype transformation associated with higher metastatic and malignant potential. Thus, we evaluated the effect of hypoxia on cell migration. We performed wound-healing assays to assess whether hypoxia would indeed stimulate cellular migration and if cell migration will differently affected POS and HMPOS cells. Under hypoxia, the migration ability was increased for both cell types (Fig. 3A–C). HMPOS cells showed increased migratory ability compared to the POS cells under hypoxia (*p*-value 0.0034).

**Figure 4:**
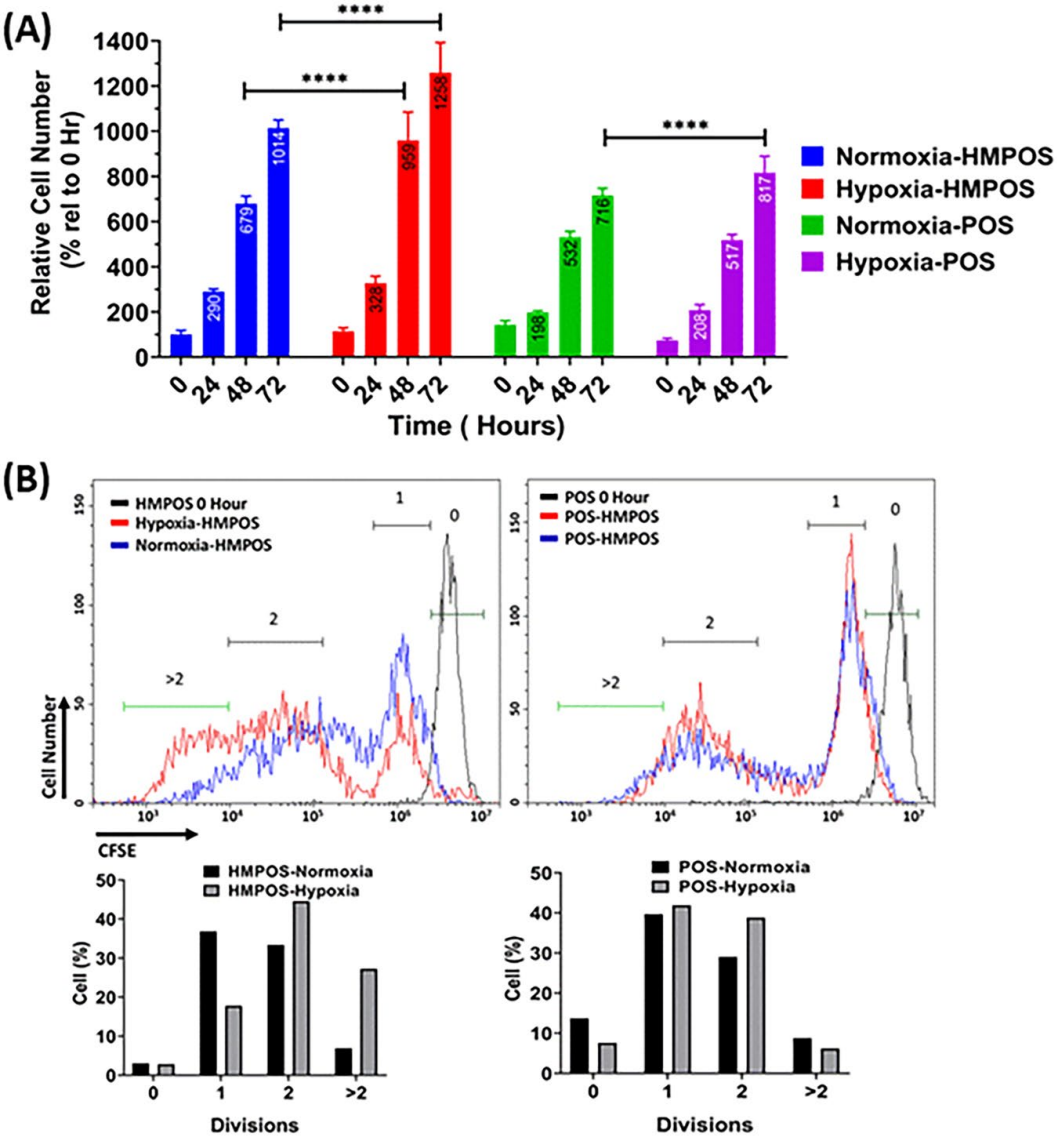
Hypoxia increases proliferation of HMPOS and POS cells. (**A**) Hypoxic condition increases proliferation rate of HMPOS and POS cells. Number of cells measured at 0, 24 48 and 72 hours as a percentage relative to HMPOS-Normoxia at 0 hours using cell viability assay. (**B**) Hypoxic condition increases proliferation rate of HMPOS cells. CFSE assay was used to measure number of cell divisions under normoxia and hypoxia. CFSE intensity after 72 hours was measured using flow cytometry displayed as histograms in upper panel. Time 0 hour sample for each cell line was used as reference for 0 division cells. Bottom panel is percentage of cells per number of divisions. Two-way ANOVA with Tukey’s multi-comparison post-test *** =  < 0.001, **** =  < 0.0001.

As the hypoxia-induced shift in protein expression also suggested a phenotype associated with enhanced proliferation, we performed cell proliferation assays (Fig. 4A,B). Hypoxia increased the proliferation rate of HMPOS cells more than two-fold after 48 hours of exposure (Fig. 4A). Hypoxia had less of an effect on the proliferation of the POS cells compared to HMPOS. However, the number of POS cells under hypoxia was greater than the number of POS cells under normoxia at 72 hours (Fig. 4A). Proliferation rate for the HMPOS cells was greater than for the POS cells but independent of hypoxia. To further confirm the role of hypoxia on the proliferation rate we used a carboxy fluorescein succinimidyl ester (CFSE) assay. HMPOS cells grown under hypoxia underwent more cell divisions, in accord to the proteome shift observed under hypoxia. More than 25% of HMPOS cells under hypoxia underwent more than 2 divisions compared with only 6% of HMPOS cells under normoxic conditions.

### Hypoxia-responsiveness of protein pathways and functional evaluations

#### Differential responsiveness of parental low metastatic and highly metastatic OS cells to hypoxia

We selected 31 hypoxia-responsive protein targets (Table 1 and Supplemental Table S4) relevant to cancer biology belonging to the following pathways: glycolysis and pentose phosphate pathway (PPP), collagen biosynthesis, reactive oxygen species (ROS) generation/redox regulation, and immunomodulation. The reason for selecting proteins that have functions associated with immune responses was based on our recent work showing that OS-derived exosomes contained higher amounts of immunomodulatory cargo compared with normal osteoblasts^24^. In addition, we added HIF1A and LOX as additional targets due to their relevance in cancer aggressiveness and adaption of ECM to promote invasion. In response to hypoxia the magnitude of protein expression changes was higher for the HMPOS cells compared to the POS cells (Table 1). This suggested differential sensitivities of these two OS cell types to hypoxic tension. To quantitatively assess this qualitative observation, magnitudes of hypoxia-induced protein differences in POS and HMPOS cells were compared. The absolute subtractions of z-scores between normoxia and hypoxia ($|{z}_{Hypoxia}-{z}_{Normoxia}|$) were calculated, which we defined as the “sensitivity score” to indicate the magnitude of hypoxia-induced changes for a specific protein. The median (1.9 SD) of the sensitivity scores for the HMPOS system shifts towards the right side of the X-scale, indicating a larger magnitude of hypoxia-induced expression level changes for the proteins detected in the HMPOS cells compared to the protein level changes observed for the POS cells (1.4 SD) (Fig. 2D). Similar trends were also observed when the z-scores of protein targets were grouped based on their involvement in cellular pathways, glycolysis (*p* = 3.03E-03), collagen biosynthesis/remodeling (*p* = 0.0126), and ROS/redox regulation (*p* = 0.14) (Fig. 3D–F).

**Table 1:** Summary of PRM-based quantification of hypoxia-responsive protein targets.

Accession Number	Gene Name	Pathway Molecular Function	*p* Value Treatment in LR	Fold Change^a^		CV (%)
				HH/NH	HP/NP	
E2R2C3	GPI	Glycolysis	9.01E-05	1.84	1.60	7.01
E2RRC9	PGK1	Glycolysis	5.25E-06	2.12	2.13	7.79
E2RT65	PGAM1	Glycolysis	1.28E-05	2.30	1.91	8.86
F1PAM3	IDH2	Glycolysis	1.63E-04	2.08	2.07	6.21
F1PAZ2	HK2	Glycolysis	8.33E-08	3.98	3.15	6.39
F1PBT3	ALDOA	Glycolysis	6.70E-07	2.69	2.73	8.29
F1PCH3	ENO1	Glycolysis	4.53E-04	1.79	1.36	4.79
F1PHR2	PKM	Glycolysis	3.81E-05	1.50	1.42	3.15
F1PTZ9	GAPDH	Glycolysis	8.58E-06	2.12	1.78	2.81
F1PVW0	LDHA	Glycolysis	2.97E-06	2.08	1.99	4.17
F1Q3S9	PFKL	Glycolysis	1.67E-06	2.38	1.89	13.85
P54714	TPI1	Glycolysis	2.10E-05	2.17	1.94	1.57
E2RLQ6	PGLS	PPP	2.00E-05	1.69	1.47	7.77
F1PE28	TKT	PPP	5.80E-02	1.45	1.16	6.88
E2RLA2	P4HA1	Collagen Biosynthesis	3.60E-06	1.98	1.96	8.63
F1P9Z8	PLOD1	Collagen Biosynthesis	4.03E-06	2.15	1.65	8.49
F1PZL5	PLOD2	Collagen Biosynthesis	5.73E-06	3.16	2.60	12.55
J9NZK5	LOX	Collagen Biosynthesis	6.90E-05	2.22	1.86	16.01
E2QYF3	AFG3L2	Oxidative Stress	8.98E-02	0.80	0.99	5.95
E2R7L1	PDIA4	Oxidative Stress	5.44E-01	1.05	1.04	4.79
E2RHG2	PRDX1	Oxidative Stress	3.05E-01	1.12	1.01	1.27
E2RNL3	PRDX4	Oxidative Stress	8.25E-04	1.48	1.30	3.04
E2RRD4	PRDX3	Oxidative Stress	2.15E-01	1.15	1.15	3.12
F1PC59	PRDX6	Oxidative Stress	6.74E-01	1.09	0.99	2.98
F1PCG4	PRDX2	Oxidative Stress	8.67E-01	0.82	1.04	7.33
F1PEN6	TFRC	Oxidative Stress	9.36E-01	1.87	1.75	18.49
F1PY21	GSR	Oxidative Stress	9.79E-01	0.70	0.71	6.60
J9P4L2	ERP29	Oxidative Stress	1.16E-01	1.18	1.19	3.28
J9P813	SIIGMAR1	Oxidative Stress	2.12E-01	1.14	1.32	7.09
E2RNW5	ERO1L	Oxidative Stress Immune Editing	6.90E-06	2.98	1.99	8.25
F1PX14	EPHA2	Immune Editing	3.91E-03	1.83	1.47	5.90
F1P7M0	NPEPPS	Immune Editing	5.04E-04	1.41	1.36	6.39
F1PZ54	HIF1A	Transcriptional Factor	1.70E-02	1.63	1.23	6.64
O18840	ACTB	House Keeping	9.84E-01	1.08	0.94	1.81

^a^HH, HMPOS cells cultured under hypoxic conditions; NH, HMPOS cells cultered under normoxic conditions; HP, POS cells cultured under hypoxic conditions; NP, POS cells cultured under normoxic conditions.

#### Hypoxia-induced upregulation of HIF1A and adaption of a Warburg proteotype

**Figure 5:**
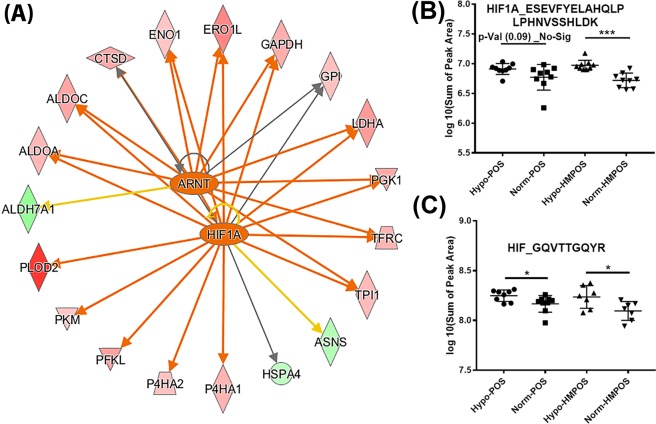
PRM-based confirmation of hypoxia-induced expression level changes of HIF1A in OS cells. (**A**) Ferris wheel of HIF1A downstream targets. The proteins at the periphery of the Ferris wheel resulted from the label-free comparative shotgun proteomic analysis. Ingenuity Pathway Analysis revealed that HIF1A and its co-translocator ARNT (located at the center of the Ferris wheel) were predicated to be activated in OS cells under hypoxia stress; (**B**,**C**) PRM-based confirmation of hypoxia-induced expression level changes of HIF1A. Peak areas of the two HIF1A signature peptides for the four groups were extracted from the PRM dataset and *p* values were calculated based on two-sample t-test. Error bars ± SEM *p < 0.05, ***p < 0.0001.

HIF1A is the central transcriptional regulator of genes associated with adaption to hypoxia. Based on the differential proteins found in our shotgun data, up-stream regulator prediction analysis, Fig. 5A, showed that hypoxia-inducible factor 1α (HIF1A, *p*-value 3.89E-13, and 5.41E-15 for POS and HMPOS cells, respectively), the aryl hydrocarbon receptor nuclear translocator (ARNT, *p* = 1.13E-14 for POS; p = 1.12E-13 for HMPOS), were activated in both POS and HMPOS cells under hypoxia. We used a PRM assay to monitor changes in expression of HIF1A which indicated a moderate increase for both cell lines (fold change 1.23 for POS and 1.62 for HMPOS, *p* = 0.017), which was confirmed by WB analysis (Figs. 5B,C, S4A). Our data is in accord with a role of HIF1A in promoting proliferation, cell invasion and aggressiveness in osteosarcoma^25^.

**Figure 6:**
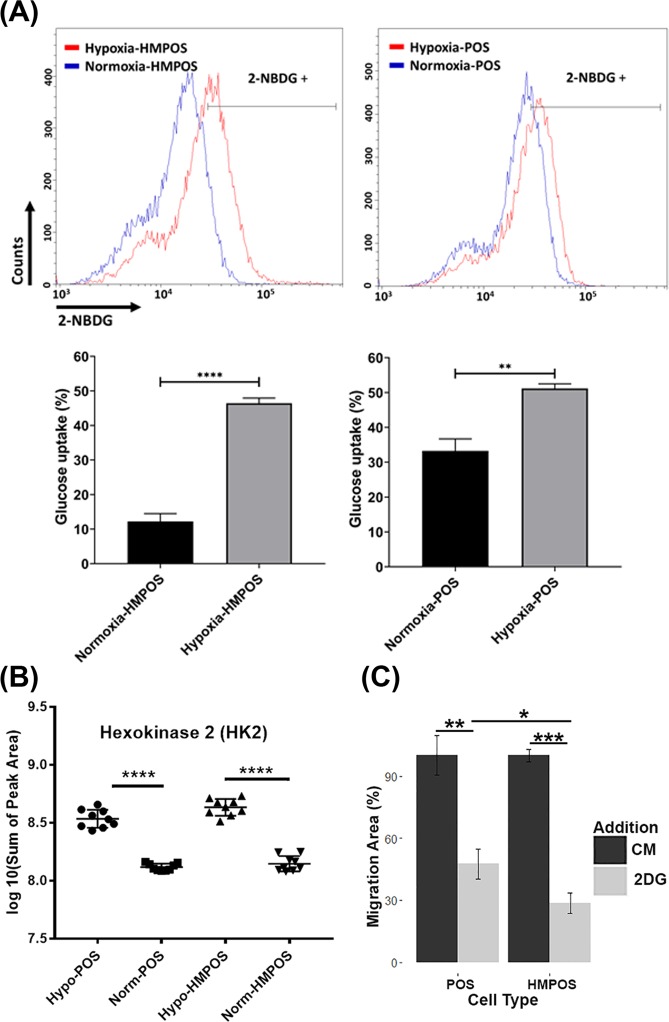
Hypoxia-induced adaption of a Warburg proteotype. (**A**) Hypoxic condition increases glucose uptake in HMPOS and POS cells. Glucose uptake was measured using 2-NBDG and quantified using flow cytometry. Upper panels, histograms indicate 2-NDBG uptake under hypoxic and normoxic conditions. Lower panel is quantification of percentage of 2-NDBG positive cells. Unpaired t-test ****P < 0.0001, **P < 0.01; (**B**) Example data obtain by PRM mass spectrometry to validate hypoxia-induced overexpression of proteins, here HK 2 in both cell types, POS and HMPOS; (**C**) Example data for using a wound-healing assay for probing the impact of hypoxia on cellular migration. Here, the HK2 inhibitor, 2DG, impedes the migration abilities of both POS and HMPOS cells under hypoxia. Error bars ±SEM *p < 0.05, ** < 0.01, ***p < 0.001, ****p < 0.0001.

The PRM data confirmed that the enzymes of the glycolysis pathway and PPP were up-regulated under hypoxia in both cell types consistent with the adaption of a Warburg proteotype and uncontrolled anabolism^26^ (Supplemental Tables S3B,C). The hypoxia-induced proteome shift was most pronounced in the HMPOS cells (Fig. 3D), suggesting an increased capability of the HMPOS cells to reprogram glucose metabolism and PPP-mediated enhanced production of reducing equivalent (NADPH) to maintain redox homeostasis. To confirm an increased demand for glucose uptake under hypoxic conditions, we performed a glucose uptake assay using 2-[N-(7-nitrobenz-2-oxa-1,3-diazol-4-yl) amino]-2-deoxy-d-glucose (2-NDBG), a fluorescent glucose analogue. Cells were grown in normoxia or hypoxia for 24 hours and then exposed to 2-NDBG for 30 minutes and analyzed for 2-NDBG uptake using flow cytometry. Hypoxia increased glucose uptake in HMPOS cells by 30% compared to a 10% increase in POS cells (Fig. 6A).

**Figure 7:**
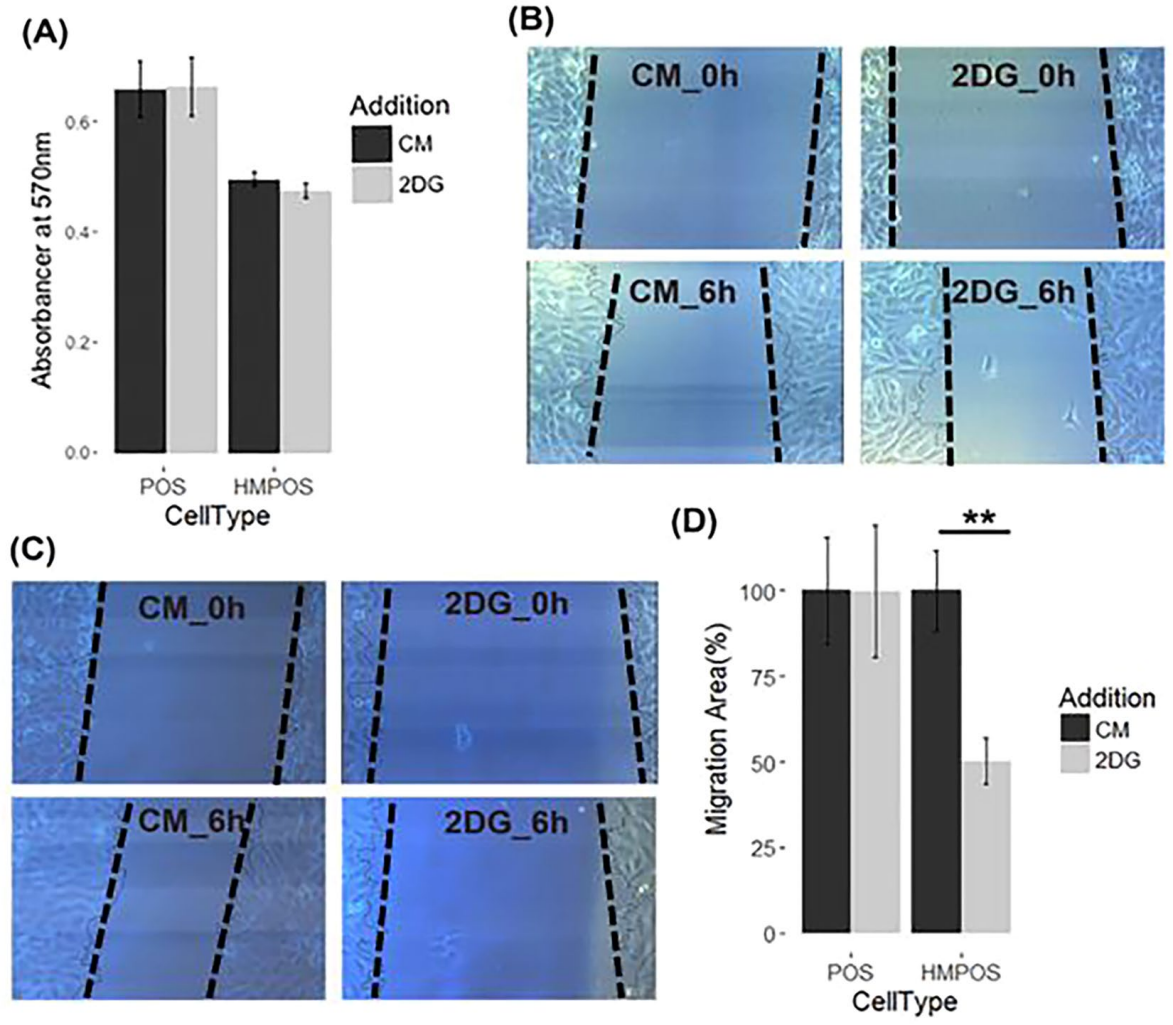
Inhibitory effect of 2DG on cellular migration abilities was hypoxia-dependent for POS cells, while hypoxia-independent for HMPOS cells. (**A**) MTT assay for cytotoxicity of 10 µg/mL 2DG on POS cells (*p* value = 0.96, df = 9.98) and HMPOS (*p* value = 0.28, df = 9.97) cultured under normoxia; (**B**,**C**) Images of wound-healing assays of POS cells (*p* = 0.988, degree of freedom (df) = 7.64) and HMPOS cells (*p* = 0.008, df = 6.56 with Welch-correction) treated with 10 μg/ml of HK2 inhibitor—2DG—for 6 hours under normoxic conditions; (**C**) 2DG inhibits the migration ability of HMPOS cells, while it has no significant effect on the migration ability of POS cells under normoxic condition. Error bars. ±SEM. **p < 0.01.

HK2 expression status has been reported to be associated with cancer cell aggressiveness including enhancing invasion, metastasis and therapeutic resistance^27–30^. Our PRM data indicate hypoxia dramatically induced overexpression of HK2 in both POS and HMPOS cells, with fold changes of 3.15 and 3.98, respectively (Fig. 6B), which was also confirmed by WB (Fig. S5B). To assess to what extent glycolysis affects the cellular migration ability of POS and HMPOS cells, 2-deoxyl-D-glucose (2DG) was used as a competitive inhibitor of HK2^31^. Under hypoxic conditions, migrations of both OS cell types were significantly inhibited by 2DG (Figs. 6C & S4C,D). The inhibitory effect of 2DG was more pronounced in the HMPOS cells compared to the POS cells (Fig. 6C). Interestingly, under normoxia the migration ability of POS cells was not significantly impaired by 2DG (Fig. 5). In contrast, 2DG significantly inhibited the migration ability of HMPOS cells even under normoxia (Fig. 7). The high dependency of the HMPOS cells on Warburg metabolism results in vulnerability to perturbation of the glycolysis pathway.

#### Hypoxia induces collagen remodeling machinery to promote OS cell migration

**Figure 8:**
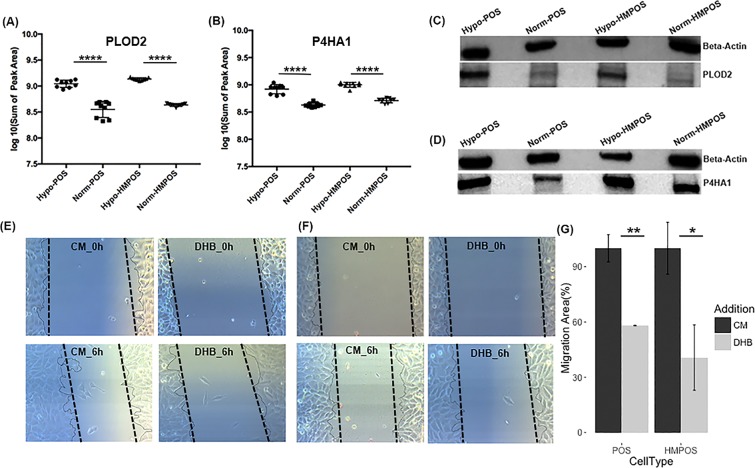
Hypoxia promotes OS cell migration via collagen biosynthesis. (**A**,**B**) The hypoxia-induced overexpression of PLOD2 and P4HA1 in both POS and HMPOS cells was confirmed by PRM mass spectrometry; (**C**,**D**) Confirmation of the hypoxia-induced increase in the expression of PLOD2 and P4HA1 in both cell types, POS and HMPOS, by western blotting; Targeted bands were cropped, and the full-length bands are shown in Supplementary Figs. S9, S10. (**E**,**F**) Images of wound-healing assays of POS cells and HMPOS cells treated with 1 μg/ml of the P4HA1 inhibitor DHB for 6 hours under hypoxic conditions; (**G**) DHB inhibits the migration abilities of both POS and HMPOS cells under hypoxia. Error bars, ±S.E.M. *p < 0.05, **p < 0.001.

Our label-free proteomic screens showed that pro-collagens and enzymes involved in collagen hydroxylation, i.e. COL5A1, COL5A2, P4HA1, PLOD1, PLOD2, were upregulated under hypoxic conditions in both cell lines. Upregulation of the key players involved in collagen biosynthesis, deposition and remodeling was confirmed by PRM assay, monitoring 16 representative peptides belonging to P4HA1, PLOD1, PLOD2, and LOX with at least 2 peptides for each protein target. Table 1 lists the proteins involved in collagen hydroxylation (PLODs and P4HA1) and crosslinking (LOX) that showed disparate increases in the two OS cell lines when cultured in hypoxic conditions. For PLOD1, which is specific for lysine hydroxylation in the α-helical or central domain of collagen, we observed under hypoxic conditions a 2.2-fold increase in the HMPOS cells and 1.7-fold increase in the POS cells compared to normoxic conditions. This significant difference in expression of PLOD1 suggests that the hypoxia-induced up-regulation is cell-type dependent. In addition, PRM analysis confirmed hypoxia-induced up-regulation of PLOD2 and P4HA1 and this trend was confirmed by WB (Fig. 8A–D). Hypoxia caused upregulation of LOX in both cell-types, but upregulation was more pronounced for HMPOS cells suggesting enhanced collagen crosslinking/remodeling capacity in line with their high metastatic propensity.

**Figure 9:**
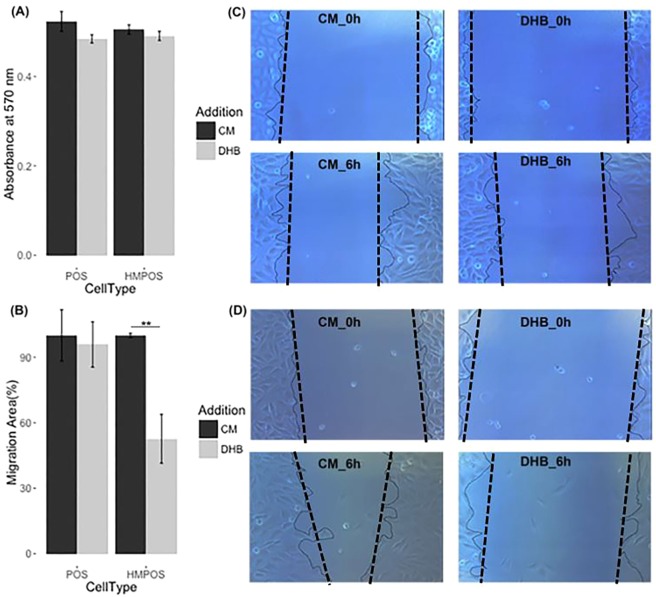
Inhibitory effect of DHB on cellular migration abilities was hypoxia-dependent for POS cells, while hypoxia-independent for HMPOS cells. (**A**) MTT assay for cytotoxicity of 1 μg/ml DHB on POS cells (*p* value = 0.15, df = 6.7) and HMPOS cells (*p* value = 0.35, df = 8.87) cultured under normoxia; (**B**) The migration ability of POS cells was not significantly inhibited by DHB *(p* = 0.4027); while the migration ability of HMPOS cells was significantly inhibited by DHB (p = 0.0067); (**C**,**D**) Images of wound-healing assays of POS cells and HMPOS cells treated with 1 μg/ml of DHB for 6 hours. Error bars. ±SEM. **p value < 0.01.

To evaluate the effects of hypoxia-induced collagen remodeling on OS cell migration, we inhibited prolyl hydroxylase activity, without affecting protein expression, by using the α-ketoglutarate analogue, ethyl 3,4-dihydroxybenzoate (DHB), as an established substrate inhibitor of the P4HA1 isoform^32,33^. We exposed both cell lines to DHB for 6 hours under either normoxic or hypoxic conditions, and then evaluated the effects of inhibition of P4HA on cellular migration using the wound-healing assay. Under hypoxic conditions, both POS and HMPOS cells exhibited decreased migration in the presence of 1 μg/ml DHB (Fig. 8E–G). The inhibitory effect of DHB on migration of POS cells was hypoxia dependent as no difference was observed under normoxia. (Fig. 9). In contrast, migration of HMPOS cells was reduced by DHB even under normoxia, suggesting a higher dependency of the HMPOS cells on collagen biosynthesis.

#### Hypoxia deregulates proteins with roles in redox homeostasis

**Figure 10:**
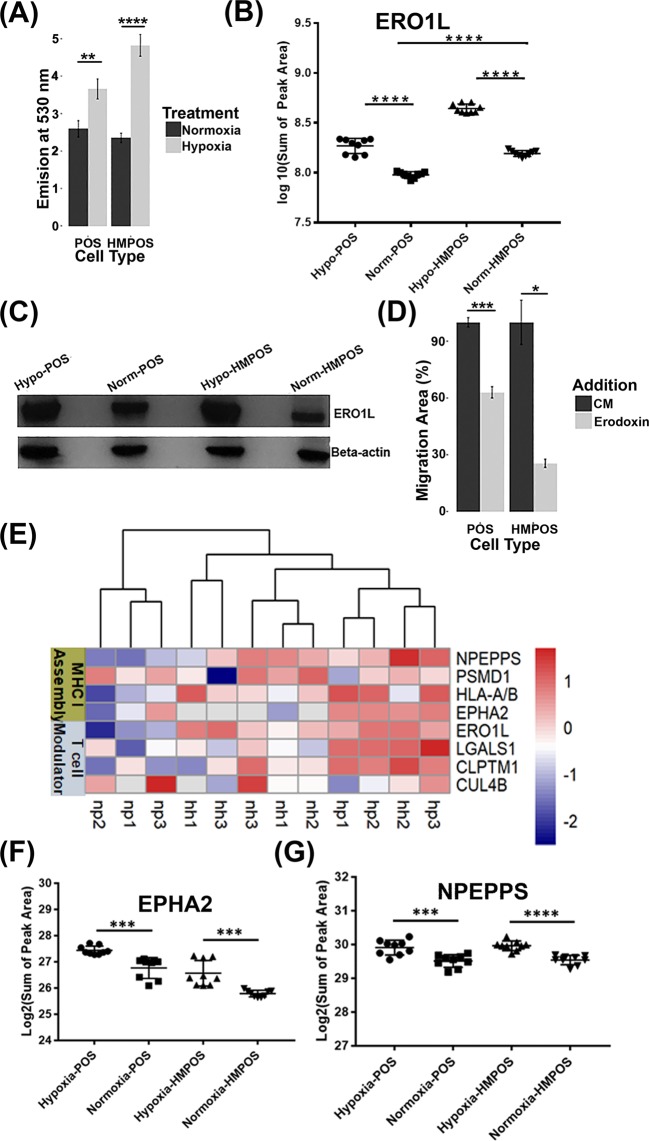
Hypoxia-induced regulation of redox homeostasis, overexpression of ERO1L and induction of cell-surface/secretory proteins associated with immunomodulation in both POS and HMPOS cells (**A**) Moderate increase of ROS levels in POS cells (*p*-value: 0.0179, degree of freedom: 6.983 with Welch-correction) and HMPOS cells (p-value < 0.0001, degree of freedom: 10) were confirmed by DCFDA assays; (**B**) PRM mass spectrometry confirms hypoxia-induced overexpression of ERO1L; (**C**) The hypoxia-induced overexpression of ERO1L was validated by western blotting; Targeted bands were cropped, and full-length bands are presented in Supplementary Fig. S11. (**D**) Erodoxin inhibits the migration abilities of both POS and HMPOS cells under hypoxia; (**E**) Heatmap visualization of relative abundances of hypoxia-responsive membrane/surface proteins related to immunomodulation; (**F**,**G**) Hypoxia-induced expression level changes of EPHA2 (**F**) and NPEPPS (**G**) were validated by PRM mass spectrometry. Error bars, ±S.E.M. **p < 0.001, ****p < 0.00001.

We found a cluster of differentially expressed proteins involved in cellular redox homeostasis, including endoplasmic reticulum resident protein 29 (ERP29)^34^, Ras-related protein R-Ras (RRAS)^35^, peroxiredoxin-1 (PRDX1), glutathione disulfide reductase (GSR), protein disulfide isomerase 4 (PDIA4), ERO1-like protein alpha (ERO1L/ERO1A)^36^, isocitrate dehydrogenase (IDH2)^37^ and 6-phosphogluconolactonase (PGLS)^38^. The deregulations of these proteins prompted us to further evaluate whether hypoxia indeed induced changes in ROS levels in OS cells. Therefore, we performed ROS/superoxide detection assays. Hypoxia led to 1.4- and 2.1-fold increase of ROS/superoxide levels as indicated by the increase of fluorescence intensity in POS and HMPOS cells, respectively (Fig. 10A). Our results are in line with the notion that both cell types adapt a proteome to counteract elevated ROS production by expressing antioxidant proteins, PPP proteins and increasing the levels of their oxidative protein folding machinery to meet demands of a highly proliferative cell population (Fig. 4A,B).

We observed upregulation of ERO1L by hypoxic treatment (Fig. 10B,C). ERO1L is an oxidoreductase in the endoplasmic reticulum and facilitates disulfide bond formation during oxidative protein folding. High levels of ERO1L have been linked to poor prognosis in many cancer types^26,36,39^. To the assess the contribution of ERO1L to the enhanced migratory ability of OS cells, we used the small molecule inhibitor Erodoxin to inhibit ERO1L activity^40^. Inhibition of ERO1L significantly reduced the migration of OS cells under hypoxia in wound-healing assays (Figs. 10D and S5). The inhibitory effect of Erodoxin was more pronounced for HMPOS cells compared to POS cells under hypoxia. This suggested that HMPOS cells rely more heavily on ERO1L under hypoxic conditions compared to POS cells (Fig. S5).

#### Hypoxia-induced upregulation of immunomodulatory proteins in OS cells

Our DDA proteomic analysis indicated upregulation of five hypoxia-responsive membrane and surface proteins: Ephrin type-A receptor 2 (EPHA2), Galectin-1 (LGALS1), HLA class I histocompatibility antigen (HLA-A/B), Puromycin-sensitive aminopeptidase (NPEPPS/PSA), 26S proteasome non-ATPase regulatory subunit 1(PSMD1) (Fig. 10E). These proteins perform functions related to membrane dynamics, cell-cell interactions and immunomodulation and thus likely contribute to the aggressiveness of OS. Expression level changes of EPHA2 and NPEPPS were further confirmed by PRM mass spectrometry (Table 1 and Fig. 10F,G). EPHA2 has been previously identified as a highly expressed surface protein in OS cells and tissue^41–43^. We found that LGALS1, a potent inducer of T-cell apoptosis^44,45^, was also significantly increased in both POS and HMPOS cells when cultured under hypoxia. LGALS1 upregulation has been reported in human OS tissue samples^46^ and is associated with metastatic and aggressive cancer cell phenotypes^47–49^. Hypoxia stimulated the MHC class I assembly based on upregulation of PSA^50^, PSMD^51^, and HLA-A/B. The hypoxia-induced upregulation of HLA-A/B is consistent with a recent study reporting elevated expression of HLA class I (HLA-A/B/C) in metastatic OS and positive association with increased PD-L1 and T-cell infiltration^52^. In contrast, a hypoxia-induced decrease in cullin 4B (CUL4B) was observed in both cell types, which has been reported to restrict myeloid-derived suppressor cells (MDSC) accumulation prohibiting the establishment of a tumor-permissive microenvironment^53^. Thus, hypoxia-stimulated up-regulation of inducers for T-cell apoptosis (LGALS1) and down-regulation of MDSCs suppressor (CUL4B) might promote an immunosuppressive microenvironment.

## Discussion

This study emphasizes the impact of hypoxia on the proteome biology of OS cells. We found that hypoxic conditions result in parental OS cells developing a similar proteotype as highly metastatic OS cells. We further report the delineation of how OS cells remodel their proteomes in response to hypoxia and develop a more aggressive phenotype. We also observed that proteins associated with immunomodulation were responsive to regulation by hypoxia which in addition may promote a microenvironment conducive to immunosuppression exasperating aggressiveness of OS.

In this study we aimed to delineate the hypoxia-driven proteotypes of two isogenic canine OS cell lines with different metastatic phenotypes: POS (low metastatic) and HMPOS (highly metastatic). To define the proteotypic responsiveness we defined a “distance” score (|${z}_{HMPOS}-{z}_{POS}$|) to quantitatively evaluate the proteome shifts exhibited by the two OS cell-types in response to hypoxia. The distance scores and principal component analysis indicated that hypoxia drives parental OS cells to remodel their proteome to adapt a proteotype similar to that one observed for OS cells with high metastatic propensity. We also introduced a “sensitivity” score ($|{z}_{Hypoxia}-{z}_{Normoxia}|$) to delineate responsiveness of distinct protein pathways and networks to hypoxia. This analysis suggested that HMPOS is more sensitive to hypoxia compared with POS, having higher magnitude of deregulation of proteins associated with glycolysis and collagen remodeling. In line with the proteomic data, functional studies of glucose uptake indicated both POS and HMPOS cells have increased glucose uptake under hypoxic conditions (Fig. 6). This further supports the reliance on the glycolysis pathway under hypoxic conditions for both metastatic and parental OS cells. A shift to glycolysis also correlated with an increased proliferation rate of both HMPOS and POS cells under hypoxic conditions (Fig. 4). This is consistent with previous studies which have reported hypoxia results in dependence on glycolysis for cell proliferation^54^. A recent glycoproteomic analysis performed on the isogenic human OS cell lines, HOS and 143B, also indicated upregulation of glycolysis/PPP proteins in the highly metastatic 143B cell line^55^. Noteworthy, a previous comparative proteomics study that focused on differentially expressed cell membrane proteins in metastatic and non-metastatic OS cells of human and canine origin showed that differentially expressed proteins associated with pro-metastatic functions were conserved between human and canine species^21^. Thus, targeting proteins involved in glycolysis in metastatic OS may inform new therapeutic strategies for canine as well as human osteosarcoma.

Under hypoxic conditions collagen biosynthesis and remodeling was upregulated based on detection of four proteins P4HA1, PLOD1, PLOD2 and LOX. Our findings are supportive of previous work that HIF-1α enhances expression of PLOD2 and promotes metastasis in sarcoma^56^. In addition, the family of collagen prolyl 4-hydroxylases (P4HAs) has been found to be overexpressed in multiple cancers, promoting cancer progression and is associated with poor clinical outcomes^57,58^. The inhibition of collagen prolyl 4-hydroxylases has been suggested as a strategy to reduce invasiveness of breast cancer^57^ and B-cell lymphoma^59^ however it remains an unexplored strategy in OS. The primary function of LOX is catalyzing crosslinking of collagens in the extracellular matrix. Increased capacity in remodeling of the extracellular matrix has been associated with highly aggressive cancers^60^. We found higher LOX levels in HMPOS cells and hypoxia induced LOX expression, both observations are supportive of an emerging role of LOX in OS. Our findings underscore that proteins associated with collagen biosynthesis and remodeling are potential targets for the development of new drugs for metastatic OS.

Although this study was designed to add mechanistic knowledge to OS biology and not designed for the discovery of OS biomarkers, markers of metastasis previously reported were detected. A recent study observed high expression of PRDX1 in lung lesions of OS patients, where PRDX1 was found to promote lung metastasis^61^. Elevated PRDX1 levels have also been detected in multiple human osteosarcoma cell lines, including MG-63, SAOS-2 and U2-OS, when compared to osteoblast cells^62^. We find PRDX1 expression levels are increased in HMPOS compared to POS cells. PRDX1 expression is enhanced by hypoxia and this sensitivity to hypoxia was particularly apparent for the HMPOS cell line. However, ambiguity in the role of PRDX1 in OS remains^62^.

In summary, our study highlights mechanistic insights into the hypoxia-driven adaptions of the proteome needed to endow OS cells with increased cell migration and proliferation abilities, attributes of aggressive cancer cell phenotypes. Our findings further suggest proteins of the glycolysis and collagen synthesis/remodeling pathways as potential targets to inhibit cell migration and metastasis in OS.

## Materials and Methods

### Chemicals and reagents

Solvents, including water and acetonitrile, were of Optima UHPLC-MS grade (Fisher Scientific (NJ, US)). Reagents used for cell cultures were purchased from Gibco Thermo Fisher Scientific (NY, US). Antibodies for Western blot assays were from Santa Cruz (CA, US). Additional details for chemicals and reagents used in this study can be found in the Supplemental Information.

### Cell lines and cell culture

Canine osteosarcoma cell lines, parental osteosarcoma (POS) and highly metastatic osteosarcoma (HMPOS), were gifts from Dr. Milan Milovancev’s lab. For all the experiment, 1 × 10^6^ cells were seeded on 75 cm^2^ flasks containing RPMI1640 with 10% FBS and 1% PS (abbreviated as CM for “complete medium”) at 37 °C using normoxic conditions (21% O_2_ and 5% CO_2_). After reaching 50–60% confluence, the cells were rinsed three times with HBSS, and fresh CM was added. The cells were then subjected to either normoxic (21% O_2_, 5% CO_2_) or hypoxic (3% O_2_, 5% CO_2_) treatments for 24 hours. Three independent biological replicates were conducted for each condition.

### Cell lysis and protein digestion

Cells were harvested by trypsinization by using 0.05% (w/v) trypsin-EDTA (Gibco^TM^, Thermo Fisher Scientific). After rinsing three times with HBSS, cells were pelleted by centrifugation at 1280 RCF (relative centrifugal force) at room temperature. Cell pellets were re-suspended in lysis buffer (50 mM NH_4_HCO_3_ containing 8 M urea and complete proteinase inhibitor cocktail), transferred to 1.5 mL Eppendorf tubes and lysed by sonication (10 × 2 s bursts) while immersed in an ice bath. Cell debris was removed by centrifugation at 15,000 RCF at 4 °C for 15 minutes.

Protein concentration was estimated using Bradford assay. Equal amount of proteins from each sample was reduced by 5 mM DTT at 56 °C for 1 hour and alkylated with 10 mM IAA at room temperature in the dark for 1 hour. The denatured proteins were precipitated by adding 4 volumes of ice-cold acetone and kept at −20 °C overnight. After centrifugation (at 15,000 RCF), the protein pellets were re-dissolved in 50 mM NH_4_HCO_3_ containing 0.1% RapiGest, digested with MS-grade trypsin using a 1:50 ratio (w/w) and incubated at 37 °C overnight. RapiGest surfactant was removed according to the manufacturer’s protocol.

### Nano-LC-MS/MS analysis and database searching

Nano-LC-MS/MS analyses were performed on an Orbitrap Fusion Lumos system (Thermo Fisher SCIENTIFIC, MA, US) coupled to nanoACQUITY UPLC system (Waters, MA, US). For each biological sample, three technical replicates were performed. Therefore, there were 36 injections for 12 biological samples belonging to four categories. The sample set was analyzed independently based on a random list generated in R. To minimize carryover between sample runs a blank was inserted between each sample. In each injection, 0.5 μg of proteome digest was loaded onto a Waters nanoAcquity UPLC 2 G V/M trap column (180 μm × 20 mm, 5 μm) for 3 min using 3% acetonitrile containing 0.1% formic acid and applying a flow rate of 5 μL/min. For subsequent peptide separation a Waters nanoAcquity UPLC BEH130 C18 column (100 μm × 100 mm, 1.7 μm) was used. After 3 minutes of desalting with 100% solvent A (HPLC-grade water containing 0.1% formic acid), a linear gradient was applied to separate the peptide mixture, starting with 3% solvent B (acetonitrile containing 0.1% formic acid) and reaching 30% in 102 minutes. The flow rate was 500 nL/minute. The nano-Electrospray emitter was held at 2000 Volts. The ion transfer tube temperature was set to 300 °C. The mass spectrometer was operated using “Top Speed” data dependent acquisition mode under XCalibur software. High-resolution mass spectral data for the peptide precursor ions were acquired in the Orbitrap mass analyzer over an m/z range of 400–1500. The Orbitrap analyzer was operated using a maximum injection time of 50 ms, a target automatic gain control (AGC) setting of 4.0e5, and a resolving power of 120,000 (at m/z 200). Low-resolution fragment ion spectra were collected in the linear dual-pressure linear ion trap using collision-induced dissociation (CID) with 30% normalized collision energy. Dynamic exclusion was employed within 60 seconds to avoid repeated sampling of the same peptide precursor ions. The intensity threshold of precursor ion selection for MS2 was set at 5.0e3 with charge exclusion of z = 1 ion.

Protein identification and quantification were performed using Proteome Discoverer (PD, version 2.0). MS^2^ spectra were searched against UniProt Canine database (Version 2015) with 25,439 entries using Mascot (Version 1.7) as search engine. The mass tolerances for precursors and fragments were set to 10 ppm and 0.8 Da respectively. Two missed cleavage sites were allowed; oxidation on methionine residues was set as a dynamic modification, while carbamidomethylation on cysteine residues was set as a static modification. Top 3 unique or razor peptides were used for protein area calculation and quantification. Decoy proteins catenated to UniProt Canine database was used to estimate the FDR. A 1% FDR cutoff was applied at both peptide level and protein level.

### Parallel reaction monitoring (PRM) mass spectrometry and data analysis

The PRM assay developed and applied in this study falls under Tier III based on the tier structure described in the guidelines for targeted MS^63^. A sequentially scheduled PRM method was applied and performed on an Orbitrap Fusion Lumos mass spectrometer coupled with the same nanoUPLC setup as described above. The same linear gradient was used for the reversed phase separation of the peptide mixtures. For target precursor ion selection, the front-end quadrupole was operated with a 2 Da isolation window. After HCD fragmentation (25% normalized collision energy), all fragment ions were pushed into the Orbitrap analyzer for m/z scanning with 30,000 resolving power at m/z 200. AGC value was set to 5 × 10^5^ using a maximum fill time of 50 ms. Representative peptide candidates for monitoring were selected using Skyline software followed by experimental assessments^64^. Peptide candidates given by Skyline (Version 3.5.0.9319) were analyzed using unscheduled survey PRMs and the generated raw files were imported into Skyline. Peptides that produced at least 3 well-aligned fragments with high mass accuracy, i.e. mass error smaller than 3 ppm, and had confirmative information in either our DDA-derived spectral libraries (dotp larger than 0.9) or SRM atlas^65^ (www.srmatlas.org) were kept as signature peptide targets. Due to the high LC stability required in the scheduled PRM method, a segmented PRM method was developed, in which the 119-minute analysis time was divided into 4 segments based on the retention time distribution of the signature peptides. A total of 139 unique peptides were selected to represent 34 proteins. Due to the restriction in cycle time proteins were grouped according to biological pathways into five groups for PRM mass spectral analysis. Five representative peptides of β-actin were also monitored to serve as loading amount controls.

PRM datasets were analyzed using Skyline software for the generation of extracted ion chromatograms and peak integration. Due to the prohibitively high costs for high purity internal standard peptides, we applied additional criteria during the data processing step to ensure correct identities of the signature peptides. Specifically, PRM raw files were searched against the UniProt Canine database using PD and the produced matched spectra files (MSF) from both PRM dataset and DDA dataset were re-imported into Skyline as reference spectral library^66^. In addition, the matched spectra were manually confirmed by comparison with the SRM atlas database. The retention times (RTs) were aligned automatically, and peak areas for peptides with wrong RTs in some injections were removed from these injections and replaced with NAs. Intensity patterns of fragment ions were inspected to ensure consistency across all 36 injections and matched to the spectral library built in Skyline. All spectral library-matched transitions with mass errors smaller than 3ppm were extracted from the PRM dataset. The exported transition results were further analyzed using R software for calculating fold changes, Welch two sample t-test, paired t-test, regression analysis and data visualizations.

### Pathway analysis and biological annotation

To better understand pathways modulated by hypoxia, a list of differential proteins (fold change > 1.2 or <−1.2) was compiled. Assessments of sequence similarities between canine and human proteins were performed using the Basic Local Alignment Search Tool (BLAST) from UniProt. The top matching entries with homology higher than 90% were selected and the human gene annotations were extracted for the subsequent bioinformatic analysis. Canonical pathway analysis, disease and function enrichment, and upstream regulator prediction were performed using IPA (QIAGEN Inc., https://www.qiagenbioinformatics.com/products/ingenuity-pathway-analysis)^67^. The HMPOS and POS systems were analyzed separately. The canonical pathways and predicted up-stream regulators were generated using as inputs the gene symbols, fold-changes and p-values derived from comparison of protein abundance levels in the normoxic and hypoxic groups.

### Experimental design and statistical rationale

For both label free and PRM analyses, a 2 × 2 experimental design was used for representing two variables with two levels for each cell type (POS and HMPOS), and treatment (hypoxia and normoxia), resulting in four groups. For each group, three independent cell cultures were used, which were designed as biological replicates. Each biological sample was injected in triplicate to generate technical replicates. All samples were analyzed in a random order to avoid any cluster effects. To assess the effect of cell type, hypoxic treatment and their possible interaction on protein expression, the label-free datasets were fitted by the following linear regression model y = β_0_ + β_1_ CellType + β_2_ Treatment + β_3_ CellType × Treatment. If the coefficient of the interaction term, β_3_, is found not to be statistically significant, the reduced linear regression model without the interaction term was fitted instead. The p-value inflation induced by multiple comparisons was also considered, and FDRs and q values were calculated and used when appropriate^68^. Z-score was calculated according to the following equation: z-score = $\frac{{x}_{i}-x}{S}$, in which *x*_*i*_ is the individual peak area for each biological sample; *x* represents the average of the peak area of all biological samples; and S is the standard deviation. Therefore, z-scores are in the unit of standard deviation with either positive sign or negative sign. The software R (version 3.2.1) was used for the calculation of z-scores. All statistical analyses and data visualizations were performed using the following R packages, ggplot2, grid, and *q*-value.

### Supplemental experimental procedures

The experimental details for western blotting and cellular assays, such as cell viability, cellular proliferation, glucose uptake, and wound healing assays, can be found in the Supplemental Information.

### Mass spectrometry data deposition

Proteomics data have been deposited to the ProteomeXchange repository (http://www.proteomexchange.org/) via PRIDE (http://www.ebi.ac.uk/pride/archive/) with the dataset identifiers PXD008986 and DIO 10.6019/PDX008986.

## Supplementary information

Supplementary information

Supplementary information2

Supplementary information.3

Supplementary information4

Supplementary information5

Supplementary information6
