# Supplementary material for: Delineation of hypoxia-induced proteome shifts in osteosarcoma cells with different metastatic propensities
Source: Sci Rep. 2020 Jan 20;10:727. doi: 10.1038/s41598-019-56878-x (PMC6971036; doi:10.1038/s41598-019-56878-x)

**SUPPLEMENTAL INFORMATION**

**Delineation of hypoxia-induced proteome shifts in osteosarcoma cells with different metastatic propensities**

*Zifeng Song^1^, Martin C. Pearce^2^, Yuan Jiang^3^, Liping Yang^1^, Cheri Goodall^4^, Cristobal L. Miranda^5^, Milan Milovancev**^4^, Shay Bracha^4^, Siva K. Kolluri^2,5^, Claudia S. Maier^1,5^*

^1^Department of Chemistry, ^2^Department of Environmental & Molecular Toxicology, ^3^Department of Statistics, ^4^College of Veterinary Medicine, ^5^Linus Pauling Institute, Oregon State University

**Running title:** Hypoxia-induced Proteome Shift in Osteosarcoma Cells

**Key Words:** Hypoxia, Metastasis, Label-free Quantification, Parallel Reaction Monitoring, Osteosarcoma

**Address Correspondence to:**

Dr. Claudia S. Maier

Professor,

Department of Chemistry

153 Gilbert Hall

Oregon State University

Corvallis, Oregon, 97331

Email: [claudia.maier@oregonstate.edu](mailto:claudia.maier@oregonstate.edu)

**Supplemental Materials and Experimental Procedures**

*Chemicals and Reagents*

Roswell Park Memorial Institute formulation 1640 medium (RPMI1640), fetal bovine serum (FBS), Hank’s Balanced Salts Solution (HBSS), penicillin/streptomycin (PS) and Halt protease, phosphatase inhibitor cocktail (100X), SuperSignal West Pico Chemiluminescent substrate and CL-XPosure^TM^ Film were purchased from Thermo Fisher Scientific (NY, US). Antibodies against HK II (ab76959), P4HA1 (ab59497), PLOD2 (ab90088), HIF1A (ab179483) and ERO1L (ab177156) were purchased from ABCam (Cambridge, MA), while anti-β-actin and anti-IgGs were from Santa Cruz (CA, US). Modified trypsin (mass spectrometry-grade; Promega (WI, US)), was used for the protein digestion. While trypsin-EDTA (Gibco^TM^ Trypsin-EDTA, Thermo Fisher Scientific (NY, US)) was used for cell harvesting and passaging. 2-Deoxy-D-glucose (2DG), ethyl 3,4-dihydroxybenzoic acid (DHB), 2',7'-dichlorodihydrofluorescein diacetate (H_2_DCFDA) and 3-(4,5-dimethylthiazol-yl)-2.5-diphenyltetrazolium (MTT) were from Sigma-Aldrich (MO, US). Erodoxin, an ERO1 inhibitor, was from Calbiochem (CA, US). Dithiothreitol (DTT), iodoacetamide (IAA) and Mini-PROTEAN TGX gels were purchased from BIO-RAD (CA, US). RapiGest SF surfactant was obtained from Waters (Milford, MA, USA).

*Cell viability assay*

The cytotoxic effect of 2DG (10 μg/mL), DHB (1 μg/mL) and Erodoxin (1 μg/mL) on POS and HMPOS cells was assessed by the MTT assay. Briefly, around 20,000 cells were seeded in each well of a 96-well plate, and 24 hours later, treated with 2DG, DHB or Erodoxin under normoxic condition for 24 hours. After incubation in 0.5 mg/mL MTT solution for 3 hours, insoluble formazan was dissolved in acidified isopropanol and absorbance at 570nm was measured on a microplate reader.

*Cell viability proliferation assays*

HMPOS and POS cells were plated at 2000 cells per well in a 96-well black tissue culture plate and allowed to adhere in 10% serum medium. After 30 minutes of plating cells number of cells for 0-hour time point was measured using Titer Glo (G7570, Promega, Madison, WI). Titer Glo was added to the wells at the assay end points 0, 24, 48 and 72 hours according to manufacturer’s protocol. Luminescence was measured using a Tropix TR717 Microplate luminometer. Percentage of viable cells was expressed relative to vehicle (100%).

*Carboxyfluorescein succinimidyl ester (CFSE) proliferation assay*

For CFSE analysis, cells were stained using 5 µM CFSE for 20 minutes at room temperature. After incubation RPMI containing 10% FBS was added to stop labeling and cells were washed twice using PBS and counted for plating. The remaining dead cells were fixed using 3.7% paraformaldehyde (PFA) as 0-hour control for 0 division population. Plated cells were cultured for 72 hours in hypoxic or normoxic conditions. Cells were harvested and fixed using PFA 3.7%. Samples were then washed, and flow cytometry analysis was performed using a CytoFLEX S flow cytometer (Beckman Coulter, Brea, CA); 10,000 events were analyzed using CytExpert software (Beckman Coulter).

*Wound healing assay*

Approximately 1×10^5^ cells were seeded into each well of a 24-well plate. After 24 hours incubation, a wound line was gently and slowly scratched on the cell monolayer with a sterile 1 μL pipette tip. The detached cells were removed by rinsing three times with HBSS. Each well of the 24-well plate was replenished with fresh complete medium with or without inhibitors and incubated for 6 hours at 37°C under hypoxic condition (3% O_2_, 5% CO_2_). Images were capture by an Axiovert 10 inverted microscope (Zeiss, Germany) equipped with a 3CCD color video camera, DXC-960MD (Sony, Japan), at two time-points, i.e. 0 hours and 6 hours. ImageJ software (version 1.49) was used for data processing of the migration images.

*Glucose uptake*

Cells were cultured in hypoxia or normoxia for 24 hours with RPMI 10% FBS and then washed thrice with PBS. 2-[N-(7-nitrobenz-2-oxa-1,3-diazol-4-yl) amino]-2-deoxy-d-glucose (2-NBDG) was added to PBS containing 10% FBS to a final concentration of 300 µM and then added to the cells for 30 minutes in either hypoxia or normoxia. After incubation cells were washed twice with PBS, stained for dead cells using Propidium Iodine (PI) (cat: P1304MP, Invitrogen) and harvested for flow cytometry analysis using a CytoFLEX S flow cytometer (Beckman Coulter, Brea, CA). Ten thousand (10,000) PI negative events were analyzed using CytExpert software (Beckman Coulter).

*Dichlorofluorescein diacetate (**DCFDA)-cellular reactive oxygen species detection assay*

A total of 10,000 cells were seeded into each well of a black 96-well plate with RPMI 1640 (without phenol red) supplemented with 10% FBS and incubated at 37°C under either hypoxic or normoxic condition for 24 hours. After washing cells with RPMI 1640 (without phenol red), 100 μL of 10 μM DCFH-DA was added into each well. After 20 minutes of incubation under either hypoxia or normoxia the DCFH-DA solution was removed by aspiration and cells were washed three times with RPMI 1640 (without phenol red). After adding fresh RPMI 1640 medium (without phenol red, without FBS) fluorescence was measured in each well using a fluorescence plate reader with a maximum excitation and emission wavelengths of 485 nm and 529 nm, respectively.

*SDS-PAGE and Western blotting*

For SDS-PAGE and Western blotting samples from three biological replicates were pooled. Same amounts of protein lysates were diluted with loading buffer to 1 μg/μL, followed by denaturation at 95°C for 5 minutes. After cooling down to room temperature, 10 μL of protein sample from each condition was loaded and separated on a 10% SDS-PAGE gel. Proteins were transferred to a nitrocellulose membrane and immunoblotted with primary antibodies against HK2 (mouse monoclonal), P4HA1 (goat polyclonal), PLOD2 (rabbit monoclonal), HIF1A (rabbit monoclonal), and ERO1L (rabbit monoclonal). Nitrocellulose membranes were probed with horseradish peroxidase (HRP) conjugated secondary antibodies. After adding HRP substrates, chemiluminescence was formed and light signals were captured on a film, which was further developed using a KODAK PRX-OMAT processor (Model M6B).

**Supplemental Figures**


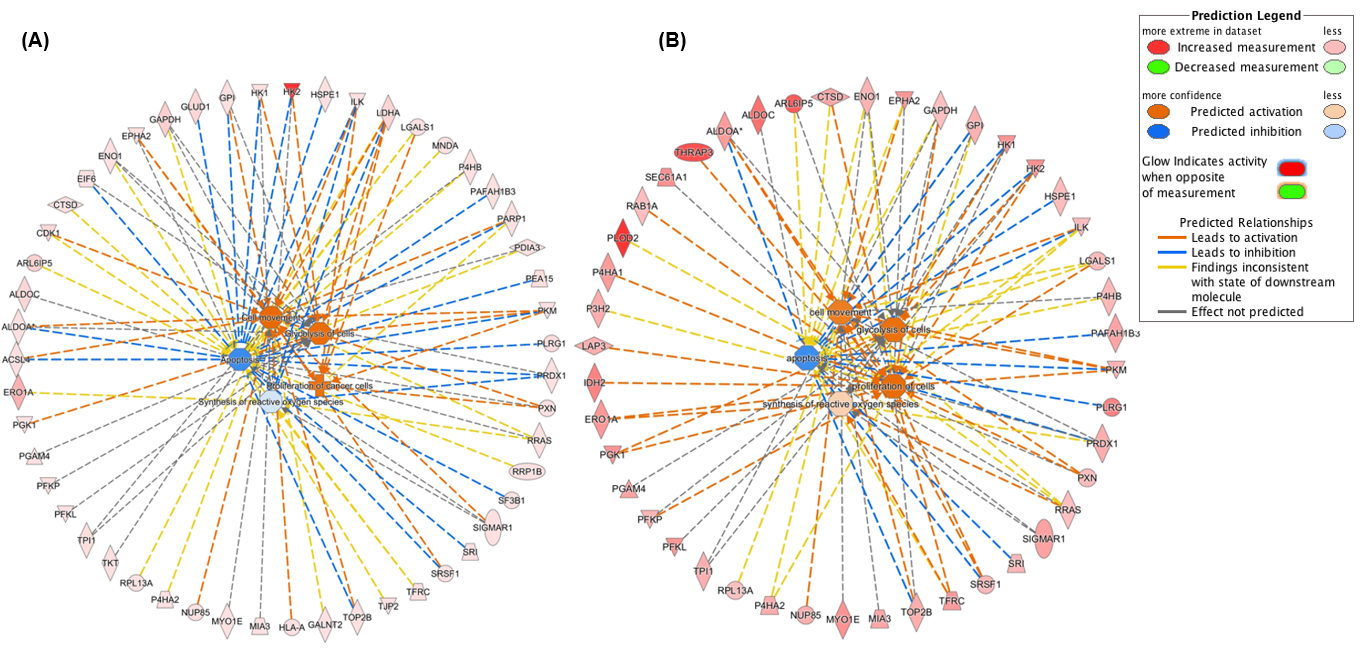
**Figure S1. Hypoxia induces proteome alterations associated with metastatic phenotype*.*** Farris wheels of hypoxia-responsive proteins that are associated with glycolysis, cellular growth and proliferation, biosynthesis of ROS and cellular movement in POS (A) and in HMPOS (B). Disease and function annotations were performed on hypoxia-responsive proteins in POS and HMPOS using IPA. Proteins associated with glycolysis, cellular growth and proliferation, apoptosis, synthesis of reactive oxygen species, and cellular movement were extracted. Relative expression levels were color-coded and visualized in figures. Red: up-regulation under hypoxia. Activity of cellular functions were predicted based on the differential protein expressions in POS and HMPOS, and activation scores were calculated. Red/Oregon: activated; Blue: inhibited.

**Figure S2. PRM-based quantification for the hypoxia-responsive protein targets are positively correlated with DDA counterparts.** Spearman’s correlation analysis was performed on PRM dataset and DDA dataset. And the correlation matrix for the Spearman’s correlation coefficients is color coded by red and green, indicating positive and negative correlations respectively. And the protein accession number ordered alphabetically on both x and y-axes.


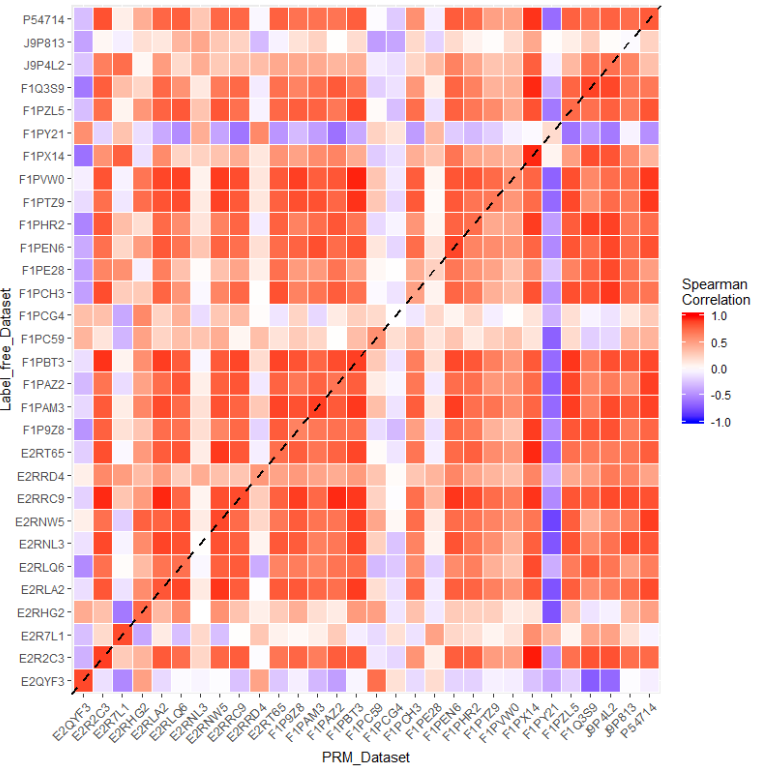


**Figure S3. Highly metastatic OS cells (HMPOS) are more sensitive to the hypoxic tension than parental low metastatic OS cells (POS).** Dot plot for normalized z-scores (calculation based on protein XIC from PRM assay) of hypoxia.


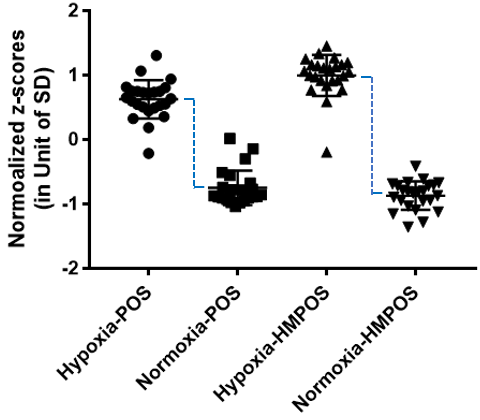


**Figure S4.** **Western blot analysis of HIF1A expression.** (A) Western blot validation of the expressions of HIF1A in POS cells and HMPOS cells grown under normoxic or hypoxic conditions. Targeted bands were cropped, and full-length bolts are presented in Supplementary Figure S7. (B) The hypoxia-induced up-regulation of HK 2 in both cells, POS and HMPOS, were validated by western blotting; Targeted bands were cropped, and full-length bolts are presented in Supplementary Figure S8. (C)-(D) Images of wound-healing assays on POS and HMPOS treated with 10 μg/ml of HK2 inhibitor—2DG—for 6 hours under hypoxic conditions


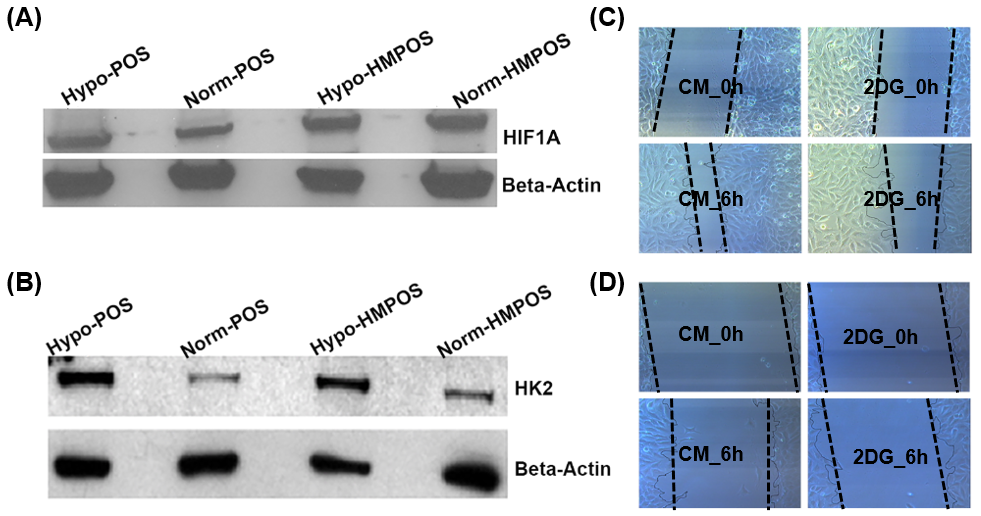


**Figure S5. Erodoxin inhibits migration abilities of POS and HMPOS cells cultured under hypoxia.** (A)-(B) Images of wound-healing assays of POS and HMPOS cells treated with 1μg/ml of ERO1L inhibitor Erodoxin for 6 hours under hypoxic conditions.

**Figure S6. The inhibitive effects of Erodoxin on migration were hypoxia-independent for both OS cell types, POS and HMPOS.** (A) MTT assay for the cytotoxicity of 1 μg/ml Erodoxin on POS( *p* value= 0.52, df= 4.93) and HMPOS(*p* value= 0.52, df= 4.21) cultured under normoxia; (B)-(C) Images of wound-healing assays on POS (*p* value= 0.0021, df= 4) and HMPOS (*p* value= 0.0052, df= 4) treated with 1μg/ml of ERO1L inhibitor, Erodoxin, for 6 hours under normoxic conditions; (D) Erodoxin inhibited the migration abilities of both POS and HMPOS under normoxia.


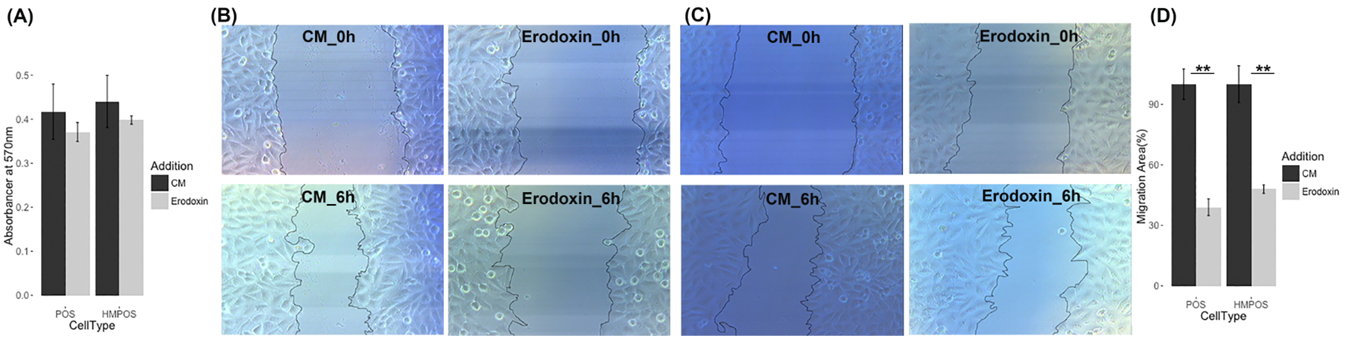


**Figure S7. Western blot-based validation of hypoxia-induced upregulation of HIF1A in POS and HMPOS.** (A)-(B) Images of full-length blots of HIF1A (A) and β-actin (B) for loading control.


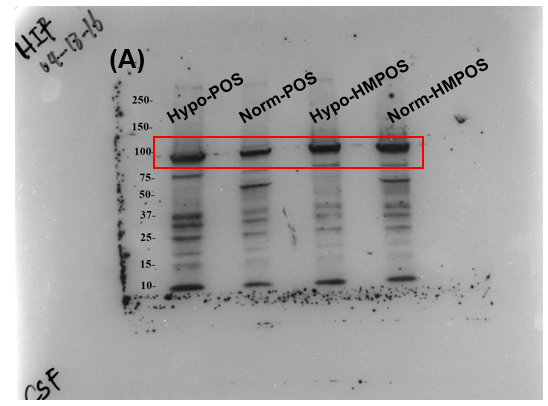


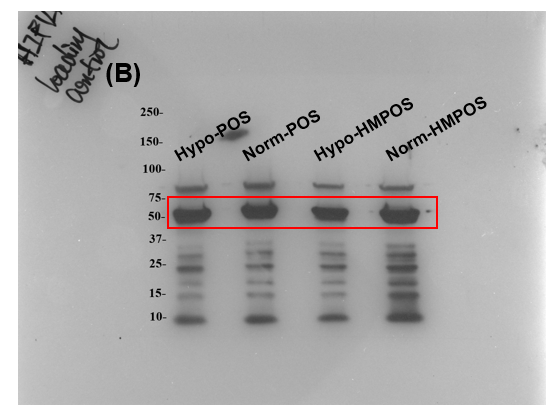


**Figure S8. Western blot-based validation of hypoxia-induced upregulation of HK2 in POS and HMPOS.** (A)-(B) Images of full-length blots of HK2 (A) and β-actin (B) for loading control.


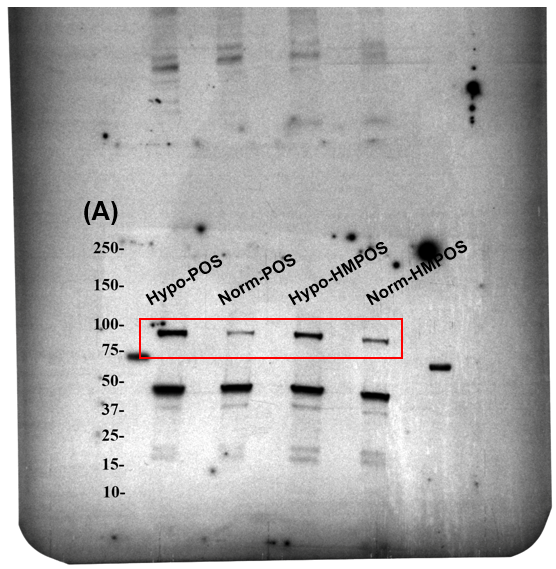


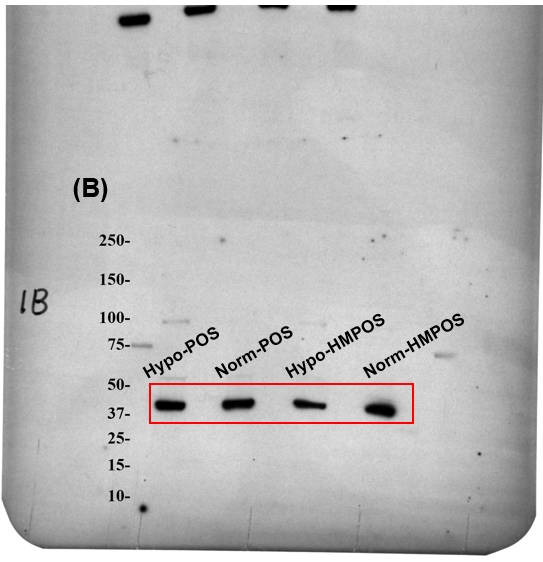


**Figure S9. Western blot-based validation of hypoxia-induced upregulation of PLOD2 in POS and HMPOS.** (A)-(B) Images of full-length blots of PLOD2 (A) and β-actin (B) for loading control.


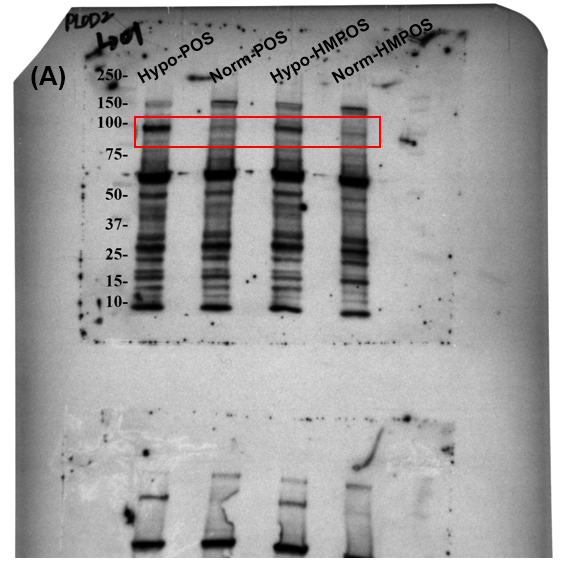


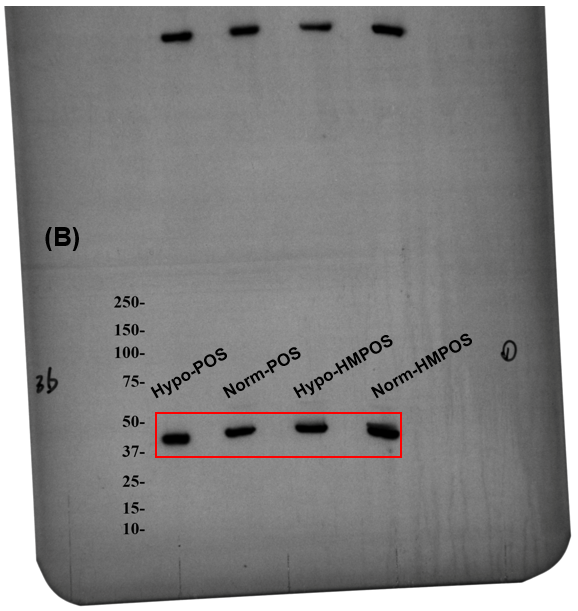


**Figure S10. Western blot-based validation of hypoxia-induced upregulation of P4HA1 in POS and HMPOS.** (A)-(B) Images of full-length blots of P4HA1 (A) and β-actin (B) for loading control.


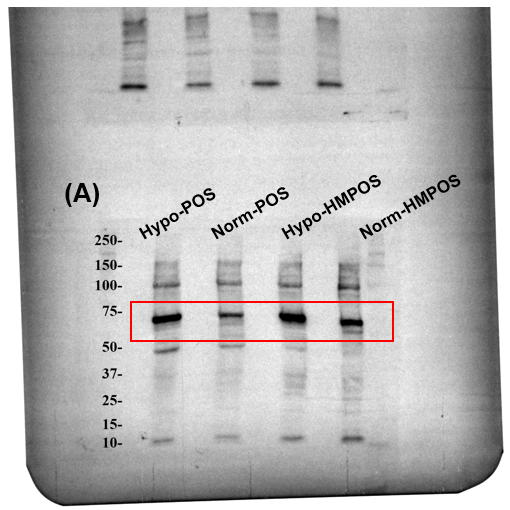


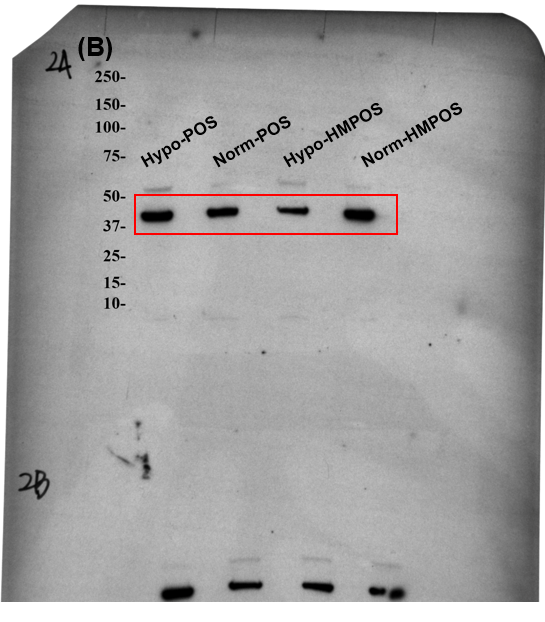


**Figure S11. Western blot-based validation of hypoxia-induced upregulation of ERO1L in POS and HMPOS.** (A)-(B) Images of full-length blots of ERO1L (A) and β-actin (B) for loading control.


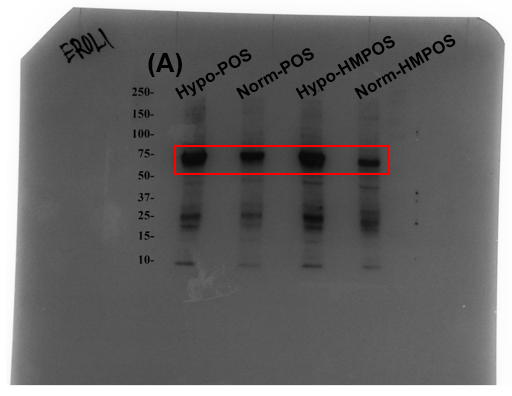


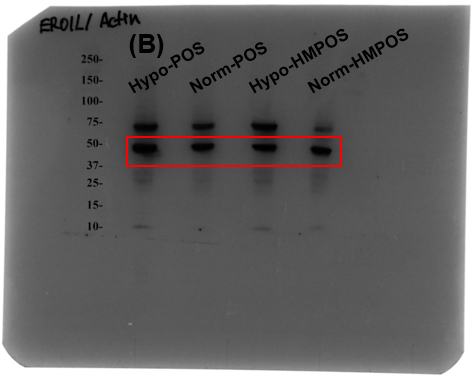

Supplement: Supplementary file 1 — Supplementary information [file 41598_2019_56878_MOESM1_ESM.docx]
