# Supplementary figures and images for: Delineation of hypoxia-induced proteome shifts in osteosarcoma cells with different metastatic propensities
Source: Sci Rep. 2020 Jan 20;10:727. doi: 10.1038/s41598-019-56878-x (PMC6971036; doi:10.1038/s41598-019-56878-x)

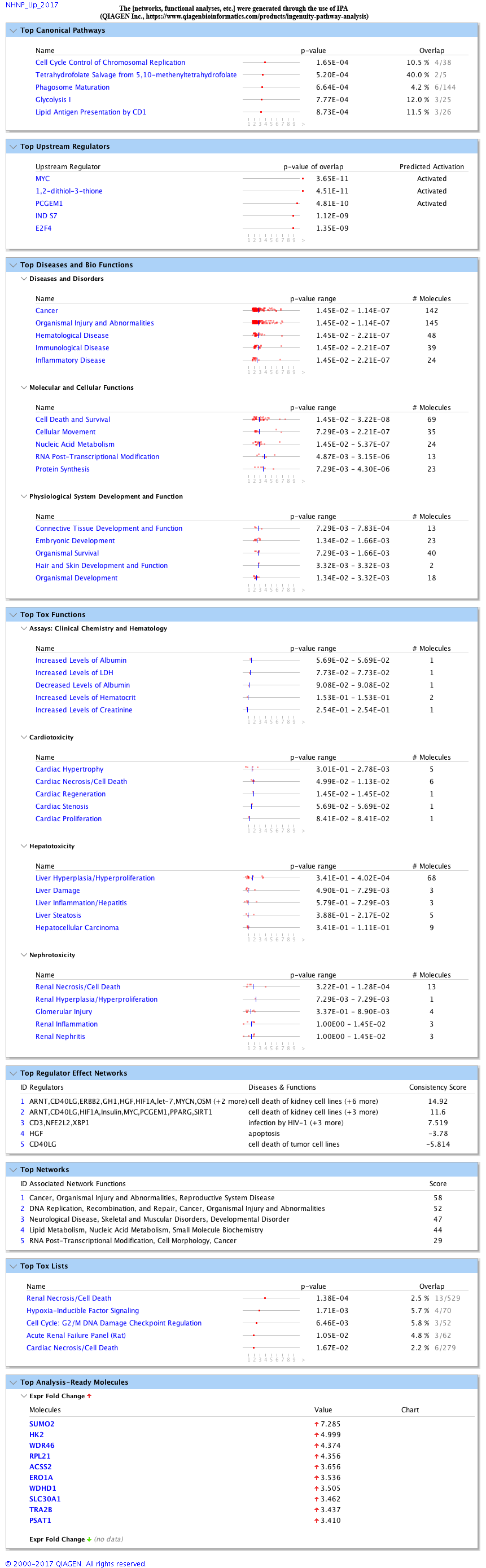

Supplement: Supplementary file 2 — Supplementary information2 [file 41598_2019_56878_MOESM2_ESM.zip › Summary1a_NHNP_Up.png]

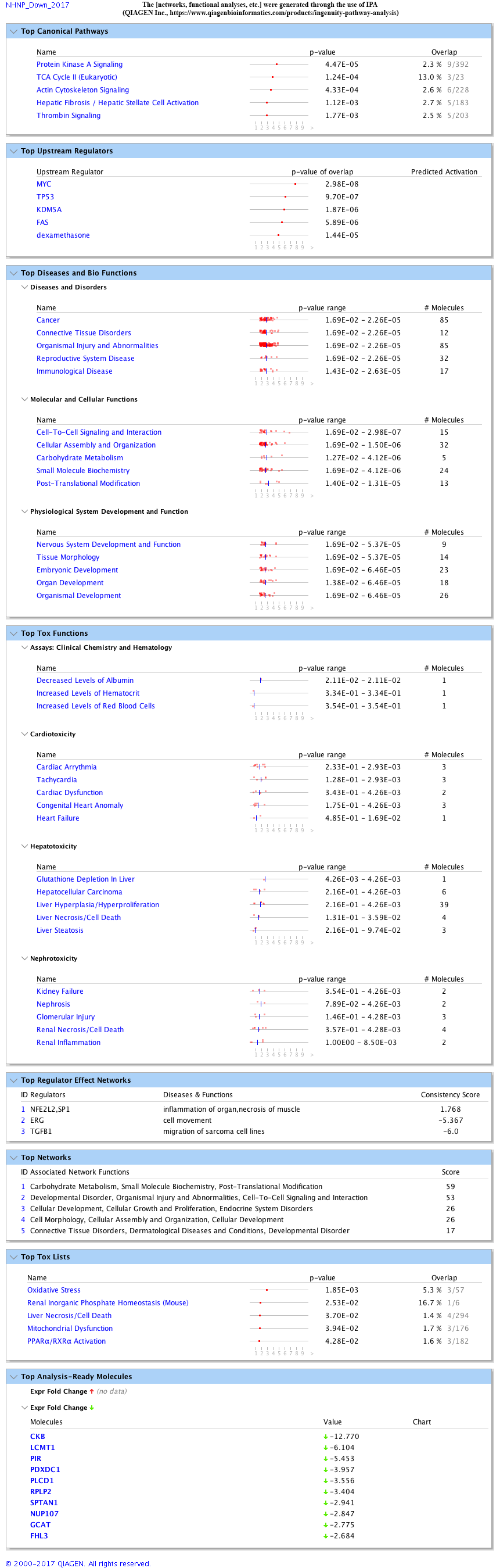

Supplement: Supplementary file 2 — Supplementary information2 [file 41598_2019_56878_MOESM2_ESM.zip › Summary1b_NHNP_down.png]

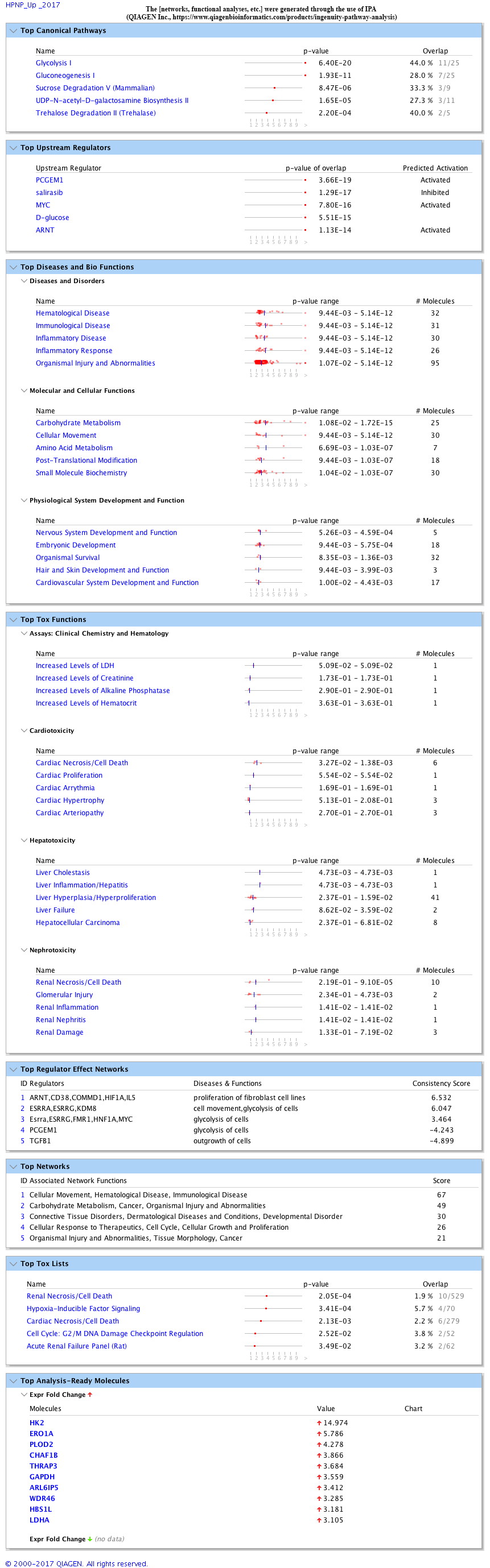

Supplement: Supplementary file 2 — Supplementary information2 [file 41598_2019_56878_MOESM2_ESM.zip › Summary1c_HPNP_Up.png]

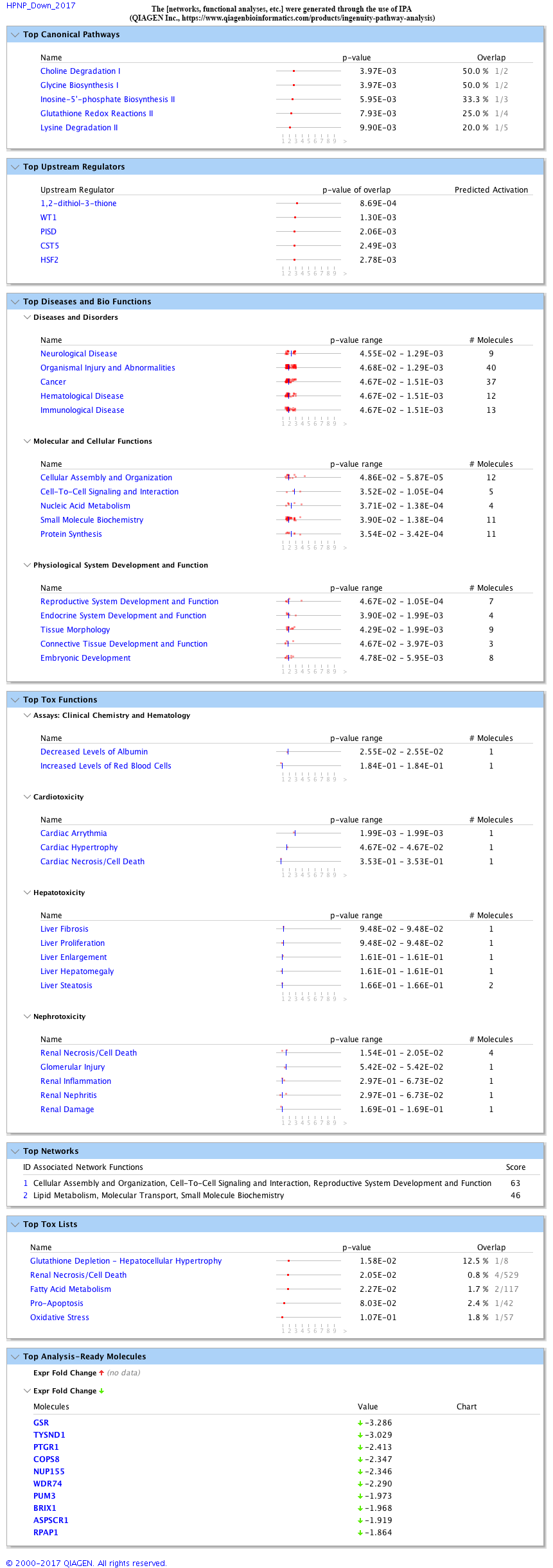

Supplement: Supplementary file 2 — Supplementary information2 [file 41598_2019_56878_MOESM2_ESM.zip › Summary1d_HPNP_Down.png]

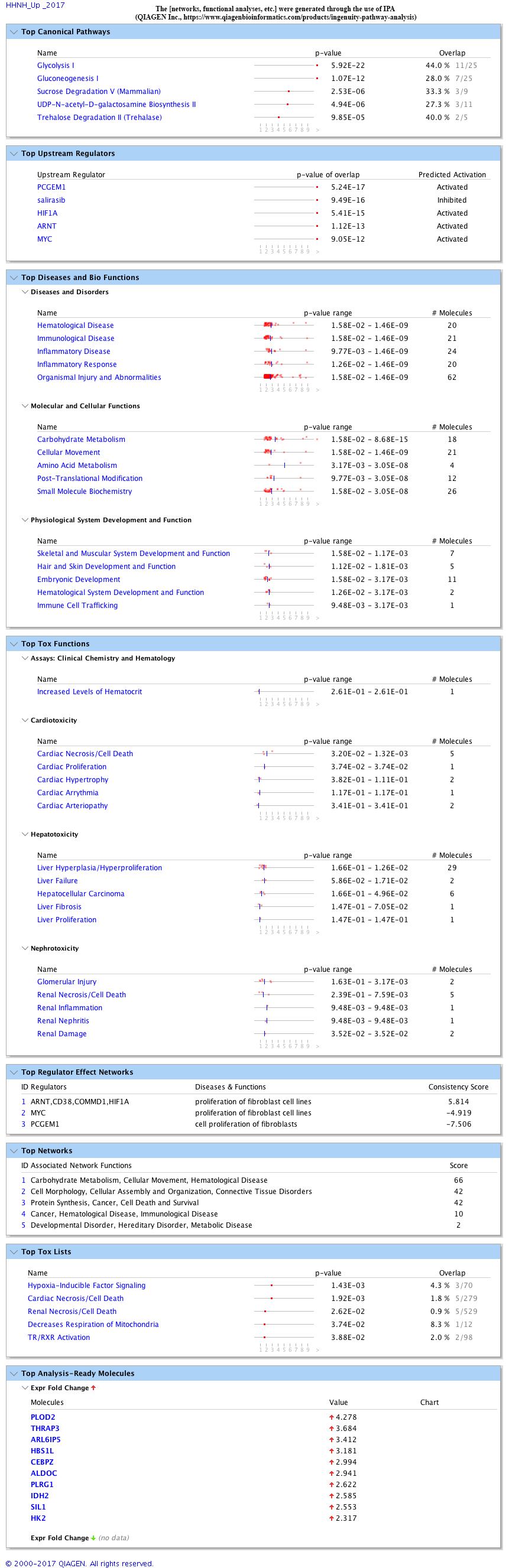

Supplement: Supplementary file 2 — Supplementary information2 [file 41598_2019_56878_MOESM2_ESM.zip › Summary1e_HHNH_Up.png]

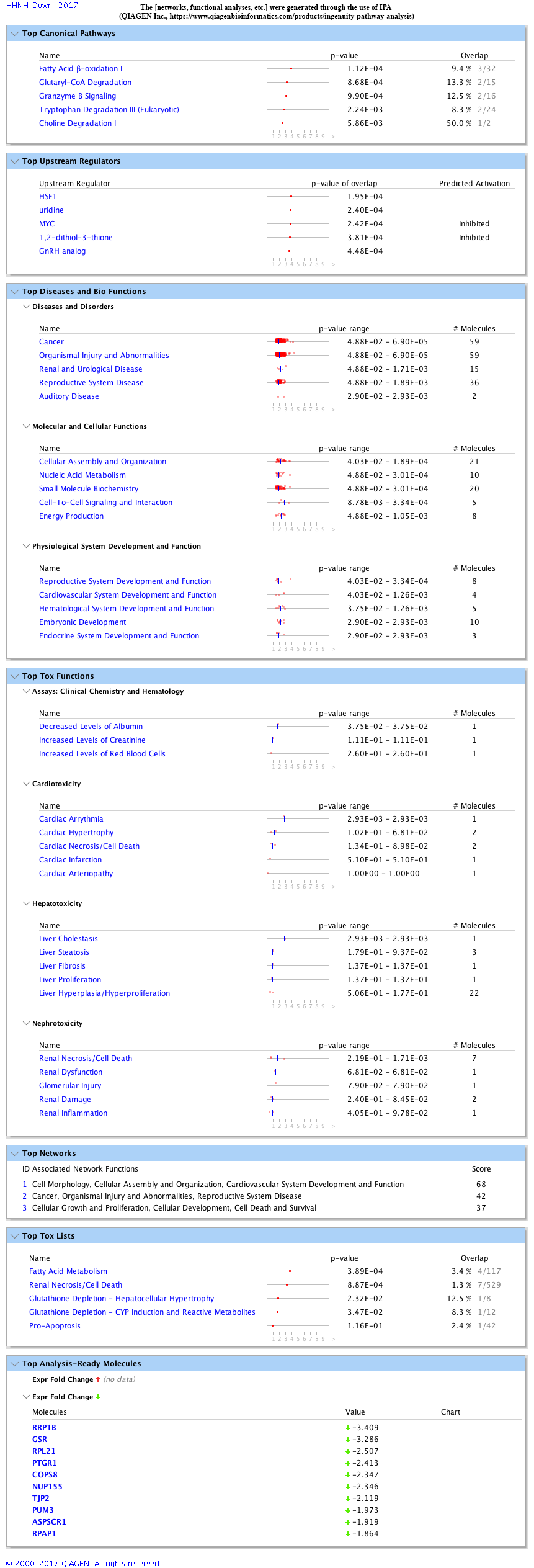

Supplement: Supplementary file 2 — Supplementary information2 [file 41598_2019_56878_MOESM2_ESM.zip › Summary1f_HHNH_Down.png]
